# Triple-M syndrome after immune checkpoint inhibitors: a systematic review of cases—early recognition is critical to survival

**DOI:** 10.3389/fneur.2026.1861948

**Published:** 2026-08-20

**Authors:** Elena Scarsi, Mehrnaz Hamedani, Sara Massucco, Edoardo Roveta, Elena Faedo, Martina Garnero, Angelo Schenone, Marina Grandis

**Affiliations:** 1 Neurology Unit Levante, Ospedale San Paolo di Savona, Savona, Italy; 2 Department of Neuroscience, Rehabilitation, Ophthalmology, Genetics, Maternal, and Child Health (DINOGMI), University of Genova, Genova, Italy; 3 Fondazione “DAVID CHIOSSONE”, Genova, Italy; 4 Neurological Clinic, AOM - IRCCS Ospedale Policlinico San Martino, Genova, Italy

**Keywords:** immune checkpoint inhibitors, myasthenia gravis, myocarditis, myositis, triple-M syndrome

## Abstract

**Introduction:**

Immune checkpoint inhibitors have markedly improved outcomes in advanced malignancies by enhancing antitumor immunity, but they may also disrupt immune tolerance and induce immune-related adverse events involving multiple organs; among these, the overlap of myositis, myocarditis, and Myasthenia Gravis (triple-M syndrome) represents a rare but potentially fatal condition with limited evidence guiding its management.

**Methods:**

We performed a systematic review of case reports and case series published until July 2025 to describe the clinical characteristics, diagnostic findings, treatments, and outcomes of patients developing triple-M syndrome after immune checkpoint inhibitor therapy.

**Results:**

The syndrome typically occurred early after treatment initiation and presented with rapidly progressive and heterogeneous features, including ocular and bulbar symptoms, muscle involvement, and cardiac manifestations; diagnosis was often challenging due to low sensitivity of routine tests, variable antibody positivity, and limited feasibility of advanced investigations, making clinical suspicion crucial. High-dose corticosteroids were the most frequently used first-line treatment, often combined with intravenous immunoglobulins or plasma exchange, although steroid monotherapy was associated with clinical worsening in some cases; emerging therapies, including targeted monoclonal antibodies and next-generation immunomodulatory agents, have the potential to provide more rapid and effective disease control. Overall prognosis remains poor, with high rates of respiratory failure, severe complications, and oncologic progression following treatment discontinuation. Triple-M syndrome is a severe and under-recognized complication requiring early diagnosis and prompt multidisciplinary management, and further studies are needed to improve diagnostic strategies and optimize treatment while preserving oncologic benefit.

## 1 Introduction

Since the approval of ipilimumab in 2011 for the treatment of metastatic melanoma, the idea of targeting the immune system instead of cancer cells has led to a paradigm shift in the oncology approach to incurable malignancies. In this last decade we have witnessed significant advancements in the development of monoclonal antibodies against cytotoxic T-lymphocyte-associated antigen 4 (CTLA-4), programmed cell death protein 1 (PD-1) and its ligand (PD-L1), leading to an expansion of therapeutic indications.

Despite the remarkable advancements in cancer therapy, disruption of endogenous immunologic tolerance mechanisms may trigger an excessive uncontrolled immune response thereby promoting the development of autoimmune manifestations (1). These treatment-specific toxicities, referred to as immune-related adverse events (irAEs), can affect any organ or system. The overall incidence of severe or life-threatening irAEs (grade ≥ 3 according to the CTCAE (Common Terminology Criteria for Adverse Events, version 5.0) ranges from 20 to 38% in patients receiving anti-CTLA-4 therapy and from 10 to 20% in those treated with anti-PD(L)-1 agents, but it can be as high as 48 to 59% in patients receiving anti-CTLA-4/PD-1 combination therapy. In general, irAEs associated with anti-CTLA-4 agents are dose-dependent and more commonly affect the dermatologic, gastrointestinal, or endocrine systems, whereas toxicities from anti-PD-1 agents appear to be dose-independent (2).

The factors influencing which patients experience immune-related toxicities, the severity of associated symptoms, and the organs involved remain largely unknown, and the specific underlying pathogenic mechanisms are poorly understood. Tissues from organs affected by immune-related toxicities often exhibit extensive lymphocytic infiltration and necrosis (3, 4). Increasing evidence suggests that B cells may also play a significant role in the pathogenesis of these toxicities, as autoantibodies have been detected in various conditions associated with immune checkpoint inhibitors (ICIs), including thyroiditis, Myasthenia Gravis (MG)/myositis, and encephalitis (5).

Neurological immune-related adverse events (N-irAEs) are reported in 0.5–2.2% of patients treated with ICIs (6) and can involve any part of the nervous system. Triple-M syndrome (myositis, myocarditis, and MG) is an extremely rare overlap syndrome characterized by the simultaneous or sequential involvement of cardiac and skeletal muscles, as well as the neuromuscular junction. Most information on its epidemiology, clinical manifestations, and diagnostic findings comes from literature reviews and case reports. Moreover, there are no established treatment guidelines for triple-M syndrome, which, although rare, is associated with high morbidity and mortality. Therefore, we systematically reviewed and summarized all cases reported in the literature.

## 2 Methods

The systematic review was conducted according to the Preferred Reporting Items for Systematic Reviews and Meta-Analyses (PRISMA) recommendations (7).

The inclusion criteria were: (i) case reports or case series; (ii) treatment with ICIs; (iii) subsequent development of triple-M syndrome (MG, myositis, and myocarditis) as complications following ICI therapy; (iv) exclusion of reasonable alternative diagnoses.

According to the PICOS model, the Participants were oncology patients who had received ICIs, the Intervention was ICI administration, the Comparator was not applicable as the reviewed papers did not provide data on controls, the Outcome was presence of triple-M syndrome, and the Study design included case reports and case series.

The PubMed, Web of Science and Scopus databases were searched on July 15, 2025, for peer-reviewed papers published from 2011 up to the date of the search, using the following search string: “(checkpoint inhibitor OR immune checkpoint inhibitor OR ICI OR anti-pd1 OR nivolumab OR pembrolizumab OR anti-pdl1 OR atezolizumab OR avelumab OR durvalumab OR anti-ctla4 OR ipilimumab) AND ((myasthenia gravis OR myasthenia OR MG OR neuromuscular junction disorder) AND (myositis OR myocarditis OR overlap syndrome OR IM3OS)).”

### 2.1 Data extraction

Search results were uploaded to Rayyan software, a web-based app designed to facilitate collaboration among reviewers during the study selection process (8). Two authors (ES, SM) independently screened the titles and abstracts. The reference lists of relevant papers were manually checked to identify additional studies that may have been missed during the database search. Any disagreements were resolved by consensus or by consulting a third reviewer (GM).

Two authors (ES, SM) independently extracted the following data from the included papers: study design, population (gender, age), oncologic diagnosis, ICI, ICI-onset (days), clinical features, autoimmunity, other paraclinical findings, instrumental diagnostic tests, therapy, prognosis, complications and comorbidities.

A systematic and descriptive analysis of the results was provided in the text and tables to summarize the characteristics and findings of the included studies.

### 2.2 Risk of bias and quality assessment

Two authors (MH, ER) independently assessed the risk of bias among the included studies using the Joanna Briggs Institute (JBI) checklist, and a third reviewer confirmed the consensus (SM). Case reports were evaluated using an 8-item JBI checklist that includes the patient’s demographic characteristics, description of diagnostic tests, medical history, treatment, current clinical condition, post-intervention clinical condition, adverse events, and the provision of takeaway lessons (9). Case series were evaluated using the 10-item JBI tool, which examines eligibility criteria, methods used to assess the condition, validity of diagnostic procedures, consecutive inclusion of participants, completeness of case inclusion, reporting of demographic and clinical data, outcome reporting, description of the clinical setting, and appropriateness of statistical methods (10). Risk of bias was quantified according to the total number of criteria met, whereby a lower score corresponded to a higher risk of bias. This assessment was not applied as an exclusion criterion, so as to allow a comprehensive synthesis of the available evidence.

## 3 Results

### 3.1 Study selection

**Figure 1:**
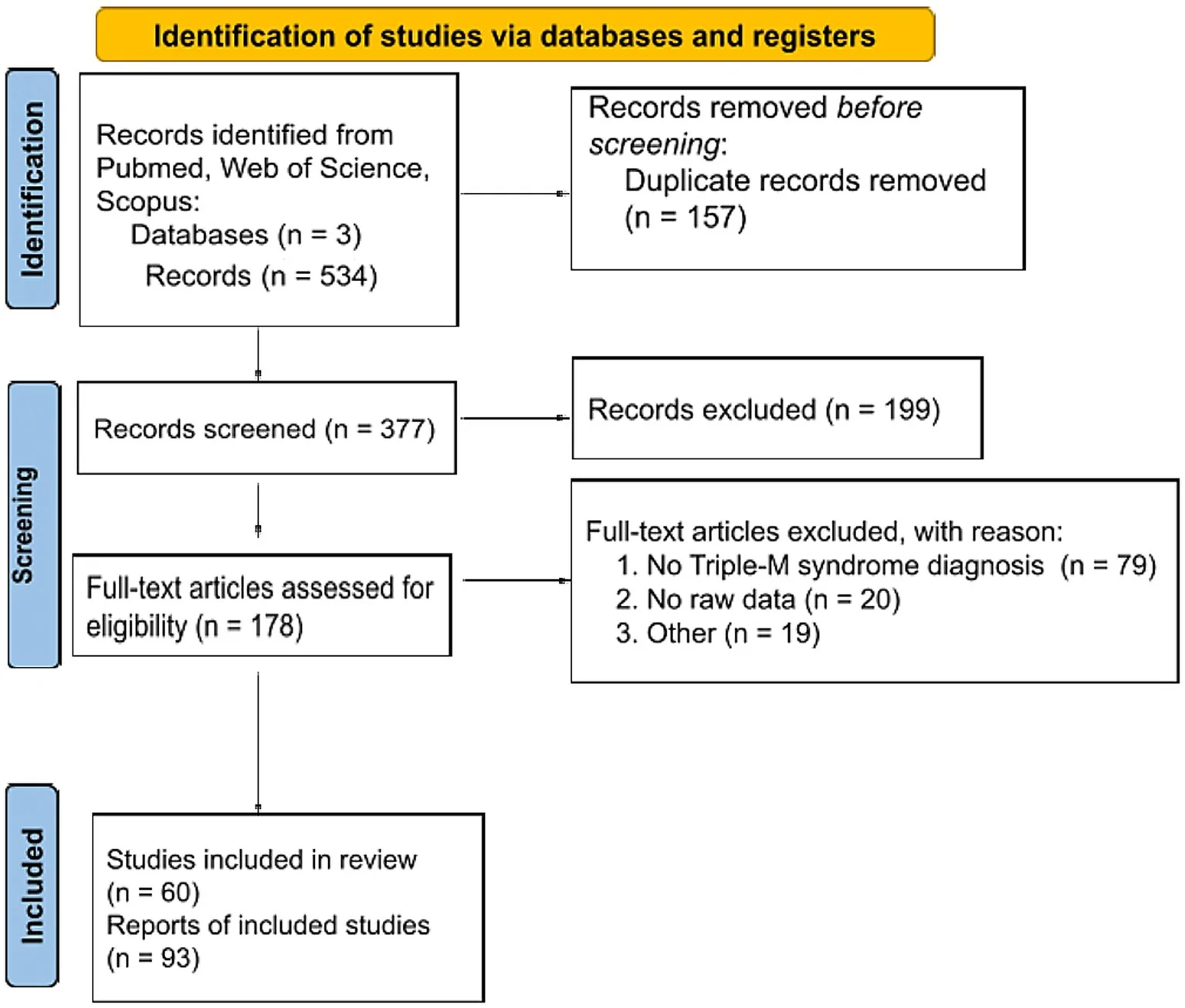
PRISMA diagram. The PRISMA 2020 flow diagram was adapted from Page et al. (7), Creative Commons Attribution 4.0,International (CC BY 4.0).

A total of 534 records were identified through the literature search, and no additional studies were found by reviewing the reference list of relevant papers. After removing duplicates (157), 377 papers were screened by title and abstract, and 178 papers were selected for full-text screening based on the predefined PICOS criteria. Notably, case reports/series describing a clinical diagnosis of the triad of myositis, myocarditis and myasthenia gravis as complications following ICI therapy were included. Cases of undiagnosed triple-M syndrome and papers lacking raw data were therefore excluded. Two authors (ES, SM) independently assessed the 178 papers selected for in-depth review. Disagreements arose with 11 articles (inter-rater agreement: 93.4%) and were resolved by consulting a third reviewer (MG). Sixty articles (with a total of 93 patients described) met the inclusion criteria cited above and were therefore included in the systematic review (Figure 1).

### 3.2 Quality of included studies

Of the 50 case reports included, 44 achieved a perfect score (8/8), while 6 received a lower score of 7/8, resulting in an overall mean score of 7.88 (Supplementary Table S1). The most significant scoring criteria were the reporting of patients’ clinical condition at presentation, the assessment methods or diagnostic tests used, and the description of the post-intervention clinical status. Among the 10 case series, 1 study attained a perfect score of 10/10, whereas 2 studies scored the lowest (3/10), leading to an overall mean score of 6.7 (Supplementary Table S2).

### 3.3 Clinical characteristics

Population characteristics are summarized in Table 1, whereas clinical presentation and cardiac assessment findings are detailed in Tables 2, 3, and Supplementary Table S3.

**Table 1:** Study characteristics.

Authors	Year	Publication type	No. patients	Oncologic diagnosis	ICI
Adeoye et al. (36)	2024	Case report	1	Lung adenocarcinoma	Anti PD L1
Aggarwal et al. (53)	2024	Case series	2	Urothelial carcinoma	Anti PD L1
				Melanoma	Anti PD1
Ahdi et al. (24)	2023	Case report	1	Renal cell carcinoma	Anti PD1
Arora et al. (25)	2020	Case series	7	Melanoma	Combination
				Melanoma	Anti PD1
				Breast cancer	Combination
				Melanoma	Combination
				Melanoma	Anti PD1
				Renal cell carcinoma	Combination
				NSCLC	Anti PD1
Bai et al. (42)	2022	Case report and LR	1	Esophageal carcinoma	Anti PD1
Baptista et al. (43)	2025	Case report	1	Colon carcinoma	Anti PD1
Basnet et al. (69)	2024	Case report	1	Melanoma	Combination
Bawek et al. (30)	2021	Case report	1	Melanoma	Anti PD1
Cham et al. (70)	2021	Case report	1	NSCLC	Anti PD L1
Chen et al. (11)	2021	Case reports and LR	1	Chordoma	Anti PD1
Duong et al. (86)	2021	Case series	1	Melanoma	Combination
Fazal et al. (32)	2020	Case report	1	Melanoma	Anti PD1
Fazel et al. (20)	2019	Case report	1	Melanoma	Combination
Fuentes-Antrás et al. (21)	2022	Case report	1	NSCLC	Anti PD1
Giglio et al. (12)	2020	Case report	1	Colon carcinoma	Anti PD1
Giovannini et al. (33)	2023	Case report and LR	1	Melanoma	Anti PD1
Hyun et al. (87)	2020	Case report	1	Thymoma	Anti PD1
Hyun et al. (88)	2023	Case series	2	Thymoma	Anti PD1
				Thymoma	Anti PD1
Jenkins et al. (3)	2022	Letter to editor	2	Melanoma	Anti PD1
				SCLC	Anti PD1
Jespersen et al. (13)	2021	Case report	1	Renal cell carcinoma	Combination
Jeyakumar et al. (47)	2020	Case report	1	Squamous cell carcinoma	Anti PD 1
Kamikawa et al. (48)	2025	Case report	1	Urothelial carcinoma	Anti PD1
Karibe et al. (44)	2025	Case report	1	Renal cell carcinoma	Anti PD L1
Kimura et al. (51)	2016	Case report	1	Melanoma	Anti PD1
Komatsu et al. (14)	2021	Case report	1	Gastric cancer	Anti PD1
Konstantina et al. (54)	2019	Case report and LR	1	Thymoma	Anti PD1
Lin et al. (22)	2023	Case report and LR	1	SCLC	Anti PD1
Lipe et al. (23)	2021	Case series	7	Thymoma	Anti PD1
				SCLC	Anti PDL1
				Urothelial carcinoma	Anti PD1
				Renal cell carcinoma	Combination
				Chordoma	Anti PD1
				Renal cell carcinoma	Combination
				Melanoma	Combination
Luo et al. (15)	2021	Case report	1	Thymoma	Anti PD 1
Marco et al. (89)	2022	Case series	4	NSCLC	Anti PD1
				Prostate carcinoma	Anti PD1
				Melanoma	Anti PD1
				NSCLC	Anti PDL1
Mariniello et al. (45)	2024	Case report	1	Lung adenocarcinoma	Combination
Muranova et al. (90)	2023	Case report	1	Thymoma	Anti PD1
Nelke et al. (37)	2023	Case report	1	Breast cancer	Anti PD1
Newman et al. (16)	2024	Case report	1	Urothelial carcinoma	Anti PD1
Portolés Hernández et al. (49)	2021	Case report	1	Thymoma	Anti PD1
Pourhassan et al. (34)	2019	Case report	1	Melanoma	Anti PD1
Rhee et al. (55)	2022	Case series	1	Melanoma	Anti PD1
Rossi et al. (6)	2023	Case series	3	Melanoma	Anti PD1
				Melanoma	Anti PD1
				Melanoma	Combination
Rota et al. (29)	2018	Letter to editor	2	Renal cell carcinoma	Anti PD1
				Renal cell carcinoma	Anti PD1
Saishu et al. (38)	2022	Case report	1	Melanoma	Anti PD1
Saito et al. (17)	2024	Case report	1	NSCLC	Anti PD L1
Sánchez-Camacho et al. (18)	2025	Case series and LR	4	Melanoma	Combination
				Melanoma	Anti PD1
				Renal cell carcinoma	Anti PD1
				Gastric cancer	Anti PD1
Sanchez-Sancho et al. (52)	2021	Case report	1	Liposarcoma	Anti PD1
Schiopu et al. (19)	2021	Case report	1	Mesothelioma	Anti PD1
Schwab et al. (39)	2022	Case report and LR	1	Urothelial carcinoma	Anti PD1
Seker et al. (71)	2023	Case report	1	Thymoma	Anti PD1
Shibuya et al. (50)	2024	Case report	1	HCC	Anti PD L1
Shirai et al. (40)	2019	Case report	1	Melanoma	Anti PD1
So et al. (41)	2019	Case report	1	Melanoma	Anti PD1
Takai et al. (91)	2020	Case report	1	Urothelial carcinoma	Anti PD1
Todo et al. (26)	2020	Case report	1	Urothelial carcinoma	Anti PD1
Veccia et al. (27)	2020	Case report and LR	1	SCLC	Anti PD1
Vermeulen et al. (28)	2020	Case series	1	Melanoma	Anti CTLA4
Wang et al. (92)	2023	Case report	1	Thymoma	Anti PD1
Wang et al. (93)	2022	Case report and LR	1	Colon carcinoma	Anti PD1
Weaver et al. (31)	2023	Case series	10	Renal cell carcinoma	Combination
				Melanoma	Combination
				Melanoma	Combination
				Melanoma	Combination
				Melanoma	Anti PD1
				Melanoma	Anti PD1
				Renal cell carcinoma	Anti PD1
				Colon carcinoma	Anti PD1
				Melanoma	Anti PD1
				Urothelial carcinoma	Anti PDL1
Xie et al. (35)	2021	Case report	1	Lung NET	Anti PD1
Xing et al. (56)	2020	Case report	1	NSCLC	Anti PD1
Yanase et al. (94)	2020	Case report	1	Renal cell carcinoma	Combination
Yin et al. (72)	2022	Case report and LR	1	Cholangiocarcinoma	Anti PD1

Anti-CTLA4, Anti–cytotoxic T-lymphocyte antigen-4 therapy; Anti PD 1, antibodies targeting programmed cell death protein 1 (PD 1); Anti PD L1, antibodies targeting programmed death-ligand 1 (PD L1); Combination, combined immune checkpoint inhibitor therapy; HCC, hepatocellular carcinoma; ICI, immune checkpoint inhibitors; NSCLC, non–small cell lung cancer; SCLC, small cell lung cancer; lung NET, lung neuroendocrine.

**Table 2:** Demographic, oncologic, and clinical characteristics of included patients.

Characteristic	*n*/*N* (%) or median (IQR; range)
Included publications	60
Publication type	
Case report	38/60 (63.3%)
Case series	10/60 (16.7%)
Case report and literature review	9/60 (15.0%)
Case series and literature review	1/60 (3.3%)
Letter to the editor	2/60 (1.7%)
Included patients	93
Male sex	58/90 (64.4%)
Age, years	70.0 (IQR 59.0–77.0; range 30.0–89.0)
Time from ICI initiation to symptom onset, days	21.0 (IQR 14.0–28.0; range 3.0–132.0)
Oncologic diagnosis	
Melanoma	32/93 (34.4%)
Lung cancer/lung neuroendocrine tumor	14/93 (15.1%)
Renal cell carcinoma	12/93 (12.9%)
Thymoma	10/93 (10.8%)
Urothelial carcinoma	8/93 (8.6%)
Gastrointestinal/hepatobiliary malignancies	9/93 (9.7%)
Other malignancies	8/93 (8.6%)
ICI therapy subtype	
PD-1 inhibitor	64/93 (68.8%)
PD-L1 inhibitor	9/93 (9.7%)
CTLA-4 inhibitor monotherapy	1/93 (1.1%)
Combination therapy	19/93 (20.4%)
Clinical presentation	
Ocular involvement	66/93 (71.0%)
Bulbar symptoms and/or dyspnea	55/93 (59.1%)
Weakness and/or myalgias	64/93 (68.8%)
Cardiac involvement	15/93 (16.1%)

ICI, immune checkpoint inhibitor; IQR, interquartile range; PD-1, programmed cell death protein 1; PD-L1, programmed death-ligand 1; CTLA-4, cytotoxic T-lymphocyte-associated protein 4.

**Table 3:** Electrocardiographic, echocardiographic, and clinical cardiac findings.

Finding	*n*/*N* (%)
Cardiac investigations	
ECG and/or echocardiographic information available	69/93 (74.2%)
Electrocardiographic findings^a^	
Cardiac electrical abnormalities (arrhythmias, conduction abnormalities, heart block)	41/69 (59.4%)
Ischemic ECG changes	11/69 (15.9%)
Echocardiographic findings^a^	
Normal/preserved left ventricular ejection fraction	28/69 (40.6%)
Hyperdynamic left ventricular systolic function	1/69 (1.4%)
Clinical diagnoses/history^a^	
Heart failure	19/69 (27.5%)
Previous atrial fibrillation	1/69 (1.4%)
Overall reporting	
Cardiac assessment reported as normal/no abnormality noted	3/69 (4.3%)
ECG and echocardiographic information not reported	24/93 (25.8%)

a Individual findings were not mutually exclusive. The denominator includes participants for whom any ECG and/or echocardiographic information was available.

Most patients were male (64.4%; *N* = 58/90), with a mean age of 67.8 ± 13.3 years (range, 30–89). Sex was not reported for three patients (6).

Ten patients had an autoimmune diathesis, including four previous cases of thymoma, two histories of previous MG, and three cases of thyroiditis. The most common comorbidities were atrial fibrillation and/or coronary artery disease (CAD) (*N* = 6, 6.4%), respiratory diseases (2 Chronic Obstructive Pulmonary Disease—COPD, 1 Obstructive Sleep Apnea Syndrome—OSAS; *N* = 3, 3.2%), type 2 diabetes mellitus (*N* = 7, 7.5%), and chronic renal failure (*N* = 2, 2.1%).

The most commonly reported symptoms were ocular (*N* = 66, 71%), bulbar (*N* = 55, 59%), cardiac (*N* = 15, 16%), and new-onset myalgias/weakness (*N* = 64, 69%). Of the 64 patients who reported new-onset weakness and myalgia as their initial symptoms, 61 had abnormal CPK levels. Of the remaining 3, one had a muscle biopsy showing findings typical of inflammatory myositis and another had a myopathic pattern on electromyography (EMG)—EMG revealed myopathic patterns in a total of 19 patients presenting with myalgia. Bulbar symptoms at onset were more common in females than in males (Chi-Square test, *p* = 0.013). In particular, 50% (*N* = 29/58) of males and 72% (*N* = 23/32) of females presented with bulbar symptoms.

Among the 55 patients who presented with bulbar symptoms, there were 34 poor prognoses (28 deaths and six cases with severe sequelae), with 25 survivors. Among the 38 patients without significant bulbar symptoms, there were 13 deaths, one case with sequelae, and 24 survivors.

Creatine kinase was markedly elevated in 96.8% of patients (*N* = 90, range 236–61.223 U/L), and troponin was elevated in 89.2% (troponin T *N* = 27; troponin I *N* = 26; unspecified *N* = 30). Of the 50 patients with available data, 48 also had transaminitis. Except for a single case of isolated elevated alanine aminotransferase (ALT) levels, all other cases involve concurrent abnormalities in both ALT and aspartate aminotransferase (AST), the latter typically with a higher value, possibly suggesting a muscular etiology. Many authors, however, acknowledge the existence of a form of hepatitis associated with triple-M syndrome—another recognized irAE that is far more common than neuromuscular complications.

There was a distinct preponderance of patients treated with anti-PD-1 or PD-L1 [PD-(L)1] drugs (*N* = 73 on monotherapy, *N* = 19 on combination therapy). Most ICI-related toxicities appeared after a median time of 21 days (IQR, 14–28.5; range, 3–132) after the first infusion. Overall, 94.6% of patients developed triple-M syndrome within 50 days of starting therapy, with 5.4% experiencing it within the first 10 days.

The main oncological diagnoses in irAE cases were melanoma (32 cases), renal cell carcinoma (12 cases), and thymoma (10 cases).

Nineteen patients had already been treated with one or more oncological therapies before starting the immunotherapy (platinum-based agents *N* = 15, gemcitabine *N* = 5, radiotherapy *N* = 4, paclitaxel *N* = 3, antimetabolites *N* = 3, doxorubicine *N* = 3, tyrosine kinase [Tyr-Kin] inhibitors *N* = 2, cyclophosphamide *N* = 2, vascular endothelial growth factor receptor [VEGF-R] inhibitors *N* = 2), while 9 patients received another treatment in combination with ICIs (platinum *N* = 5, paclitaxel *N* = 4, antimetabolites *N* = 3, VEGF-R inhibitors *N* = 2, radiotherapy *N* = 1, doxorubicine *N* = 1, cyclophosphamide *N* = 1, Tir-Kin inhibitors *N* = 1).

### 3.4 Serology and neurophysiology

Of the 75 patients tested, 50 (66.7%) were positive for anti-AChR antibodies, including 6 with previously known anti-AChR antibody positivity (Table 4, Supplementary Table S4). One patient was positive for anti-MuSK antibodies, whereas 24 (32%) were seronegative. Anti-striatal muscle antibodies (anti-titin and anti-ryanodine receptor) were assessed in 22 patients and were positive in 18 (81.8%) and negative in 4 (18.2%).

**Table 4:** Serological and laboratory findings.

Characteristic	*n*/*N* (%) or median (IQR; range)
Included patients	93
MG autoimmunity	
MG-related antibody positivity	51/75 (68.0%)
AChR antibody positivity, overall	50/75 (66.7%)
Previously known AChR antibody positivity	6/50 (12.0%)
MuSK antibody positivity	1/75 (1.3%)
Negative MG autoimmunity	24/75 (32.0%)
Not determined	18/93 (19.4%)
Anti-striated muscle antibodies	
Positive among tested	18/22 (81.8%)
Negative among tested	4/22 (18.2%)
Not determined	71/93 (76.3%)
CPK	
CPK elevation	90/93 (96.8%)
CPK level, U/L (available in 86/90 cases)	5,961.5 (IQR 2895.0–8933.8; range 236.0–61223.0)
Troponin	
Troponin elevation	81/93 (87.1%)
TnT only, among elevated cases	25/81 (30.9%)
TnI only, among elevated cases	24/81 (29.6%)
Both TnT and TnI reported	2/81 (2.5%)
Elevated troponin, unspecified assay	30/81 (37.0%)
No troponin elevation	12/93 (12.9%)
Troponin level, ng/L (available in 77 cases)	2,350.0 (IQR 760.0–6470.0; range 42.0–90, 580.0)

MG, myasthenia gravis; AChR, acetylcholine receptor; MuSK, muscle-specific kinase; CPK, creatine phosphokinase; IQR, interquartile range; U/L, units per liter; TnT, cardiac troponin T; TnI, cardiac troponin I; ng/L, nanograms per liter.

Seven patients showed *de novo* co-positivity for anti-AChR and anti-striated muscle antibodies (3, 11–15). Among the remaining patients with positive anti-striated muscle antibodies, anti-AChR and anti-MuSK antibodies were negative in 5 cases (16–19) and were not tested in 6 cases (20–23).

We collected data from 37 neurophysiological studies, including electromyography (EMG). The most common finding was a myopathic pattern, characterized by polyphasic, short-duration, or low-amplitude motor unit action potential with early recruitment (*N* = 24). The distribution of the abnormalities was specified in 9 cases. Among these, abnormalities involved the proximal muscles of all four limbs in 3 patients, the proximal muscles of upper limbs in 3 patients, both proximal and distal muscles of all four limbs in 2 patients, and were limited to the gastrocnemius muscles in 1 patient. Three patients had completely normal results. Repetitive nerve stimulation was performed in 25 patients: of these, 9 (36.0%) had a decremental response, one (4.0%) had borderline results, with a possible decrement between 5 and 10%, and 15 (60.0%) had negative results. Among patients with altered results, facial nerves were tested in two cases, upper limbs in two cases, and both facial nerves and upper limbs in two cases; the remaining four cases had no specified testing site. Among the 15 patients with negative repetitive nerve stimulation, 11 (73.3%) showed myopathic changes on EMG.

Among the 51 seropositive patients (anti-AChR or anti-MuSK positive), 19 underwent EMG; of these, 6 had a positive repetitive stimulation test (including the borderline case). Among the 24 seronegative patients, 15 underwent EMG; of these, 2 had a positive repetitive stimulation test (3, 24–28).

In 2 cases, MG-related autoantibodies were not assessed, but repetitive stimulation test showed a decremental response (29). Nine seronegative patients did not undergo EMG (16, 18, 19, 25, 30, 31). Fifteen patients underwent neither antibody testing nor EMG (20, 21, 23, 31–35). However, in 4 of these cases (21, 23), biopsy data from cardiac and/or skeletal muscle suggested an immune-mediated process.

### 3.5 Second-line tests

#### 3.5.1 Advanced imaging

A few patients underwent neuroimaging investigation (Table 5, Supplementary Table S5), including 8 muscle magnetic resonance imaging (MRI) scans (16, 20, 36–41) and 13 cardiac MRI scans (3, 11, 25, 37, 42–45). Common findings included muscle edema, hyperintense on T2-weighted sequences, and patchy late gadolinium enhancement. Data on the topographical distribution of skeletal muscle abnormalities were limited. When reported, abnormalities involved the lower limbs, particularly the proximal muscles, in 6 cases; in the remaining 3 cases with available information, peri-optic tissues and paraspinal muscles were affected. With regard to cardiac MRI, it enabled a confirmed diagnosis of myocarditis in 7 cases (in addition to one probable case and one possible case) according to the 2008 modified Lake Louise Criteria (46). In one patient, cardiac muscle was also studied using fluorine-18 fluorodeoxyglucose positron emission tomography (18F-FDG-PET), which demonstrated increased glucose metabolism (11). In five cases, cardiac MRI findings were inconclusive; it should be noted that three of these patients underwent the scan after starting treatment, which may have contributed to false-negative results.

**Table 5:** Diagnostic investigations and diagnostic pathway.

Characteristic	*n*/*N* (%)
Included patients	93
Tissue biopsy/autopsy	
Tissue biopsy and/or autopsy performed	21/90 (23.3%)
Muscle biopsy	11/90 (12.2%)
Cardiac biopsy	8/90 (8.9%)
Autopsy	3/90 (3.3%)
No raw data available for biopsy assessment	3/93 (3.2%)
Cardiac MRI	
Cardiac MRI performed	13/93 (14.0%)
Confirmed cardiac involvement on MRI	7/13 (53.8%)
Probable cardiac involvement on MRI	1/13 (7.7%)
Inconclusive cardiac MRI	5/13 (38.5%)
Muscle MRI	
Muscle MRI performed	8/93 (8.6%)
Muscle edema/abnormal muscle MRI	8/8 (100%)
Lower-limb muscle involvement	6/8 (75.0%)
Paraspinal muscle involvement	2/8 (25.0%)
Peri-optic involvement	1/8 (12.5%)
EMG / neuromuscular junction testing	
EMG testing performed	37/93 (39.8%)
Myopathic EMG pattern	25/37 (67.6%)
NMJ testing performed	25/37 (67.6%)
NMJ abnormality/decrement reported	9/25 (36.0%)
Borderline NMJ abnormality	1/25 (4.0%)
Negative NMJ testing	15/25 (60.0%)
No EMG or NMJ abnormality reported	6/37 (16.2%)
Diagnostic/treatment sequence	
Empiric therapy (before exams)	16/43 (37.2%)
At least one exam (EMG or MRI or biopsy) performed before therapy (diagnostic work-up first)	27/43 (62.8%)
Sequence not reported	50/93 (53.8%)

“First EMG, post MRI,” “first MRI, post EMG,” “first EMG, post MRIs and biopsy,” and “first EMG, post MR” were grouped under diagnostic work-up performed before treatment. EMG, electromyography; MRI, magnetic resonance imaging; NMJ, neuromuscular junction.

#### 3.5.2 Histology

Twenty-three patients underwent histological investigations (Table 5, Supplementary Table S5). Specifically, 7 patients underwent endomyocardial biopsy (14, 23, 47–50), 10 underwent muscle biopsy (3, 22, 28, 35, 37, 39, 41, 45, 51, 52), and one patient underwent both (15). Biopsy findings were similar across all cases, showing lymphocytic infiltration with a predominance of CD3+, an increased CD8/CD4 ratio, and necrotic changes (especially in the endocardium). Immunohistochemistry revealed CD45+, CD3+, CD8+ T lymphocyte infiltration and myophagocytosis (CD68+). Additionally, three post-mortem examinations (12, 21, 33) described necrotic changes with CD8+ T-lymphocyte and histiocyte infiltrates, and myophagocytosis (CD68+) in the myocardium and in the striated muscle.

Among the 56 patients who underwent diagnostic investigations, empiric therapy was administered before completion of the diagnostic work-up in 16 cases. In this subgroup, cardiac MRI confirmed myocarditis in 2 of 5 patients, all 8 EMG studies showed abnormalities, and all 4 biopsies were abnormal. Nine patients survived, either with or without sequelae.

Conversely, 27 of 56 patients underwent diagnostic investigations before treatment initiation. In this subgroup, cardiac MRI confirmed myocarditis in 5 of 8 patients, 20 of 22 EMG studies showed abnormalities, and all 10 biopsies were abnormal. Twenty patients survived, either with or without sequelae.

In the remaining 13 of 56 cases, the temporal relationship between treatment initiation and diagnostic testing was not specified.

### 3.6 Treatment and outcomes

High-dose intravenous corticosteroids were administered to all patients (*N* = 93), while 81.7% (*N* = 76) received an additional standard therapy, including intravenous immunoglobulin (IVIg), therapeutic plasma exchange (PLEX), or thymoglobulins (Table 6, Supplementary Table S6). Monoclonal antibodies (infliximab, abatacept, tocilizumab, eculizumab) were used in 14 cases. One or more immunosuppressants (mycophenolate mofetil, rituximab, tacrolimus, everolimus, cyclophosphamide) were attempted in 26 cases as second or third-line add-on therapies.

**Table 6:** Treatment strategies, outcomes, and complications.

Characteristic	*n*/*N* (%)
Included patients	93
Treatment strategies	
Corticosteroids	93/93 (100%)
IVIg, plasma exchange, and/or thymoglobulin	76/93 (81.7%)
Additional immunosuppressants	26/93 (28.0%)
Monoclonal antibody therapy	14/93 (15.1%)
Therapy mode	
Escalation therapy	66/93 (71.0%)
Combination therapy	16/93 (17.2%)
Steroid monotherapy	11/93 (11.8%)
Outcome/prognosis	
Recovery with successful discharge	41/93 (44.1%)
Sequelae at follow-up	11/93 (11.8%)
In-hospital death	41/93 (44.1%)
Complications	
Any reported complication	54/93 (58.1%)
Respiratory failure	45/93 (48.4%)
Septic shock	2/93 (2.2%)
Upper gastrointestinal bleeding	2/93 (2.2%)
Immune-related pneumonitis	2/93 (2.2%)
Immune-related thrombocytopenia	1/93 (1.1%)
Heparin-induced thrombocytopenia	1/93 (1.1%)
SIADH	1/93 (1.1%)
Multiorgan failure	1/93 (1.1%)
Hospital-acquired pneumonia	1/93 (1.1%)
Abdominal sepsis	1/93 (1.1%)
Hyperkalemia with cardiac arrest	1/93 (1.1%)
Pneumonia	1/93 (1.1%)
Renal failure	1/93 (1.1%)
Rhabdomyolysis	1/93 (1.1%)
Immune-related thyroiditis	1/93 (1.1%)
Diarrhea	1/93 (1.1%)
Erythema multiforme	1/93 (1.1%)
MG relapse	1/93 (1.1%)

Complications and cardiac-specific therapies were not mutually exclusive. Outcome was based on the main prognosis column; post-discharge deaths due to cancer progression or unrelated causes reported in follow-up notes were not counted as in-hospital deaths. CI, immune checkpoint inhibitor; IVIg, intravenous immunoglobulin; MG, myasthenia gravis.

Only 11 patients received monotherapy (corticosteroids in all cases), with recovery observed in 3 cases.

First-line high-dose corticosteroid monotherapy led to marked exacerbation or *de novo* onset of myasthenic symptoms in 18 cases, requiring escalation to IVIg or PLEX. This resulted in respiratory failure in 18 patients and death in 10 cases, with a mortality rate of 55.5% (12, 14, 16, 17, 19–21, 25, 27, 37, 42, 43, 52–55). In these cases, toxicity occurred 3-30 days after exposure to ICIs.

The most commonly used regimen was escalation therapy (*N* = 66 cases), usually consisting of initial corticosteroid treatment (*N* = 61) followed by additional therapy because of inadequate clinical response. Mortality in this group was 43.9% (29/66). An upfront combination regimen, defined as corticosteroids plus at least one additional therapy from the beginning, was used in 16 cases, with a mortality rate of 25.0% (4/16). Steroid monotherapy was used in 11 cases and was associated with a mortality rate of 72.7% (8/11). The prognosis of triple-M syndrome was poor, with an overall mortality rate of approximately 44% (*N* = 41).

Respiratory failure occurred in 48.4% of patients (*N* = 45/93), and almost half of these patients died (*N* = 26/45). Furthermore, among survivors, recovery was often incomplete and required long periods of hospitalization and rehabilitation (11 cases of neurological sequelae among the 42 patients with follow-up data available).

On the other hand, 9 patients who survived the complication subsequently experienced progression of oncologic disease (13, 18, 19, 28, 31, 36, 44, 56), while one patient developed recurrent triple-M syndrome after exposure to a second ICI (22).

Follow-up data after the complication are incomplete and heterogeneous, ranging from 1 month to a maximum of 1 year of observation; in addition to the proportion of neurological sequelae (14 cases, ranging from residual AChR antibody positivity alone to dependence on mechanical ventilation), 10 cases of disease stability or partial tumor response following discontinuation of ICI are described.

## 4 Discussion

### 4.1 Clinical features

Our data suggest a greater vulnerability to neuromuscular toxicity in men and in patients with preexisting autoimmune disease (57); however, females appear to be at higher risk of developing severe bulbar symptoms at onset.

Patients with triple-M syndrome may present with a wide range of symptoms, varying from mild to severe, and the diagnosis can be easily overlooked in the early stages; moreover, the clinical course is often rapidly progressive. Although the diagnosis is often insidious, certain distinctive features should raise suspicion of triple-M syndrome and prompt the clinician to initiate diagnostic investigations.

Ocular symptoms, particularly ptosis and diplopia, are the most common presenting features. Compared with non-ICI-related MG, these symptoms are characterized by less fatigability during exertion. Furthermore, ICI-induced myositis affects the extra-ocular muscles more frequently than other immune-mediated myopathies.

The percentage of cases with bulbar onset is higher than that in MG outside the context of ICI exposure (59% vs. 21-23%) (58).

Respiratory compromise is often multifactorial and may result from myasthenic fatigability of the accessory thoracic muscles, purely myositic involvement of the diaphragm (12, 22), or heart failure associated with myocarditis. Although ICI-associated myositis is not typically associated with interstitial lung disease, as seen in some forms of dermatomyositis and anti-synthetase syndrome, this condition may coexist within the broader spectrum of systemic irAEs, further complicating the diagnostic workup.

Myalgia and/or easy fatigability/exercise intolerance are common but highly nonspecific symptoms. Because oncologic patients frequently experience easy fatigability due to the complications of underlying malignancy, including chronic pain, stress/anxiety, and poor quality sleep, among others, fluctuations in motor performance (evidenced on examination as give-away weakness) can be difficult to detect and monitor. In our population, these were symptoms that had recently developed in relation to the patients’ underlying conditions and were severe enough to require urgent medical attention.

The poor response to acetylcholine inhibitors is multifactorial. We have explained how the generalized weakness in patients with advanced cancer and the coexistence of myositis can make improvements in fatigue less apparent. The literature describes also that the lack of response to pyridostigmine in Myasthenia caused by ICIs may be due to massive infiltration of CD8+ cytotoxic T cells, which leads to physical destruction of the neuromuscular junction rather than simple receptor blockade (59, 60). Finally, in one case, pyridostigmine exacerbated concomitant myocarditis, causing atrioventricular block (61).

Myocarditis, which was clinically evident at onset in only 15 patients, may present with palpitations, chest pain, acute or chronic heart failure, or arrhythmias, and may be associated with pericarditis and pericardial effusion. In some cases, myocarditis may follow an indolent course, manifesting only as mild ventricular dysfunction (62).

The diagnosis of definite myocarditis is challenging, as cardiac MRI is not always readily available, and patients are often too clinically unstable to undergo invasive examinations. According to Bonaca et al. (63), probable myocarditis may be defined by cardiac MRI findings consistent with myocarditis and/or a clinical syndrome suggestive of myocarditis, or elevated cardiac markers, or electrocardiogram (EKG) evidence of myo-pericarditis; alternatively, new wall-motion abnormalities on echocardiography accompanied by a compatible clinical syndrome and elevated cardiac markers or EKG evidence of myo-pericarditis may fulfill the diagnostic criteria for probable myocarditis.

Most ICI-related toxicities present early in the course of treatment, usually after two or three cycles, depending on the treatment protocol. Notably, 5% of patients developed complications subacutely, within the first 10 days after starting immunotherapy.

As expected, melanoma was the most common underlying malignancy (*N* = 32), given that it was the first historical indication for these drugs. Even more remarkable was the high number of treated thymoma cases (*N* = 10), a tumor naturally linked to an increased risk of autoimmunity. It should be emphasized that these therapies are generally used for advanced stages of cancer, and they often represent second- or third-line options in patients with no or insufficient response to first-line therapies. The rationale for using ICIs in thymoma is based on PD-L1 overexpression and the high presence of infiltrating T cells, which justify blocking the PD-1/PD-L1 pathway (64). In our population, the choice of anti-PD(L)1 drugs is confirmed in all cases of thymoma.

### 4.2 Serology and neurophysiology

The frequency of anti-AChR antibody positivity was lower than that reported in non-ICI-associated MG (66.7% vs. 80-90%), supporting the notion that the diagnosis of ICI-related MG remains primarily clinical. Although these antibodies must be interpreted in the oncologic setting, and their pathogenic role has not yet been fully clarified, the rate of positivity for anti-striated muscle antibodies appears high (18/22 tested patients), notwithstanding the small sample size. A recent expansive review of performance in clinical service demonstrates that anti-striated muscle Abs are neither specific nor sensitive in predicting malignancy or neurologic phenotypes of MG (65).

Bedside electrophysiology was performed infrequently (37 of 93 patients), likely due to technical challenges in patients with rapidly progressing disease. The most common finding was a myopathic pattern associated with spontaneous pathological activity at rest and reduced recruitment, consistent with infiltrative and inflammatory damage to muscle fibers. The high rate of negative results after repetitive nerve stimulation despite clinical features highly indicative of MG, along with the low rate of antibody positivity, further supports that the diagnosis remains predominantly clinical and that first-line diagnostic tests have limited sensitivity. Given the frequent coexistence of myopathic alterations on EMG, it can also be speculated that these changes may affect the performance of the repetitive stimulation test, potentially masking a coexisting neuromuscular junction disorder (66); the extensive use of pyridostigmine may also have been the cause of unreliable EMG results (18). Finally, there is the possibility that some patients may have severe myositis without impairment of the neuromuscular junction, but with only “MG-like” symptoms that may stem from the myositis-specific predilection for the oculomotor and respiratory muscles (67).

### 4.3 Advanced imaging and histopathology

Second-line tests were performed in a minority of cases, as they require logistical planning and time, both of which are often limited in an unstable and rapidly progressing clinical condition.

MRI is sensitive in revealing early edema within muscle fibers. Special attention should be paid to the extraocular muscles, as ICI-related myositis typically involves this area, presenting with unique clinical and imaging features (68).

Cardiac muscle often demonstrated preserved left ventricular systolic function and ejection fraction when assessed by transthoracic echocardiogram as part of first-line evaluation (3, 18, 24, 26, 31, 32, 35, 40, 44, 50, 56, 69–72). Recent specific studies have shown that ventricular function may be preserved in 65% of cases at baseline, particularly when symptoms of myositis and myasthenia gravis coexist (73).

Triple-M syndrome is an immune-mediated disease with an inflammatory basis, as confirmed by biopsy findings that were consistent across different tissues. In a 2020 study, Shelley et al. (74) reported that although their patients with post-ICI myositis had histopathology similar to necrotizing myopathy, none of them tested positive for anti-HMGCR or anti-SRP antibodies; the author speculated that the homogeneous phenotype of oculo-bulbar involvement in these iatrogenic myositis cases might be the result of a yet-to-be-discovered antibody. Although biopsy remains the gold standard for diagnosing this and other inflammatory conditions (75), its invasiveness and the time required to arrange it make it impractical during the acute phase, as confirmed by the small number of cases in our population.

We have collected the data (presented below), but the initial hypothesis that tests performed after empirical treatment yield false negatives cannot be confirmed. Further studies with larger case series are needed to better understand the optimal diagnostic and therapeutic approach for triple-M syndrome and thus provide definitive and standardized guidelines.

### 4.4 Treatments and outcomes

In the absence of standardized treatment recommendations, high-dose intravenous corticosteroids have been the most widely adopted first-line approach, often followed by an additional parenteral therapy (IVIg, PLEX, or thymoglobulins).

The frequently rapid and tumultuous course of triple-M syndrome raises concerns about the effectiveness of classic immunosuppressants, which typically require weeks to months to achieve therapeutic benefit. Meanwhile, these treatments can expose vulnerable patients with cancer, who are often receiving multiple concomitant medications, to potentially severe side effects.

There may be a stronger rationale for using faster-acting monoclonal antibodies, such as infliximab and tocilizumab, which work by suppressing inflammatory hyperactivation and are already used in other neurological conditions (e.g., cytokine release syndrome associated with CAR-T therapy for hematologic malignancies, neuro-Behçet disease, and neurosarcoidosis) (76, 77). Tocilizumab is also currently under investigation as a maintenance, steroid-sparing therapy in non-ICI-related MG (78, 79). By binding CD80/CD86, CTLA4 agonists (abatacept) act on antigen-presenting cells and lead to global T-cell anergy with limited off-target effects and reverse pathways activated by ICI; their use must be limited in time to preserve antitumor beneficial effects. Additionally, ruxolitinib, a JAK (Janus-Kinase) inhibitor that impairs T-cell activation via blockade of pro-inflammatory cytokines, has a synergistic effect with abatacept (acting downstream in the immunological synapse) (80).

In our study, rituximab was grouped with conventional immunosuppressants, despite being biologically a monoclonal antibody; this decision was based on its slow onset of action, which makes it more similar to older-generation immunosuppressants in terms of disease-modifying activity. In patients with anti-AChR antibody positivity, complement blockade may represent a rational therapeutic strategy, supported by the detection of complement deposits in the analyzed muscle biopsy samples (37). An Italian study reported the use of eculizumab guided by anti-AChR antibody positivity, allowing for the continuation of ICI treatment while resolving the ongoing neuromuscular complication (81).

Neonatal Fc receptor (FcRn) inhibitors have also been proposed as a potential therapeutic option. Through their “pharmacological plasmapheresis” mechanism, these agents may reduce circulating autoantibody levels and could be particularly relevant in this clinical context (82).

The prognosis for Triple-M syndrome is typically poor, given the rapid and unpredictable clinical progression and the diagnostic challenges associated with this condition. When respiratory failure occurs, it is difficult to reverse, and mortality approaches 50%.

In addition, our study identified 18 cases of exacerbation of myasthenic symptoms following corticosteroid monotherapy, resulting in the need for ventilatory support and poor outcomes. It has already been hypothesized in other scientific articles that the well-known paradoxical worsening of MG with high-dose steroids (83) could also be present in this context of ICI complications (84). Based on this, we strongly recommend caution when using corticosteroid monotherapy in these patients, even when myasthenic symptoms are not yet evident.

Furthermore, although these are clinically and numerically heterogeneous groups, we can observe a trend indicating that mortality is higher in the cohort receiving escalated therapy or monotherapy. We suggest that, in severe cases, it is advisable to initiate combination therapy with multiple drugs—especially fast-acting ones—immediately.

Finally, neuromuscular toxicity is generally classified as grade ≥3 and leads to discontinuation of immunotherapy. In our review, discontinuation of ICIs was associated with progression of the underlying malignancy in nine patients who survived Triple-M syndrome, five of whom subsequently died. Further studies and larger case series are needed to enable earlier diagnosis and more effective treatment strategies, with the goal of allowing continuation of immunotherapy when feasible and ultimately improving oncological outcomes. In the absence of standardized guidelines at present, the ASCO/ESMO oncology recommendations should be taken into account; although these are based on clinical cases and retrospective studies of limited scope, they provide a diagnostic and treatment algorithm for each category of irAEs (76).

We compared our data with those reported in a recent systematic review on the topic (85). We agree on the limitations due to the sample size and the heterogeneity of the data provided by the analysis of case reports and case series. We also agree that this complication is underreported in the literature and that the expansion of oncological indications for the use of ICIs will lead to greater awareness of this condition. However, based on our data, we believe we can offer some recommendations regarding the low sensitivity of first-line diagnostic tests and, above all, a warning regarding steroid monotherapy and escalating treatment regimens. We reach similar conclusions, emphasizing that early diagnosis is essential, and to that end, it is important to rely on a multidisciplinary team to address the diagnostic and therapeutic challenges posed by this syndrome.

## 5 Conclusion

The diagnosis of Triple-M syndrome is challenging because of limited awareness, heterogeneous clinical presentation, limited feasibility of invasive testing in critically ill patients, and the suboptimal sensitivity of commonly used diagnostic tools. Further studies are needed to find sensitive and reliable disease biomarkers.

In the presence of clinical suspicion of triple-M syndrome, particular caution is warranted when initiating first-line high-dose corticosteroids as monotherapy, as they may precipitate a myasthenic crisis: almost all guidelines—which are primarily focused on cases of myositis or myocarditis—recommend starting with high-dose steroids in cases of severe ICI toxicity, raising the question of whether a combination therapy of steroids and another fast-acting drug (IVIg/PLEX, monoclonal antibodies) might be more appropriate.

Conventional immunosuppressants with slow onset of action may have limited utility in this rapidly progressive condition. Therapies with faster effectiveness, such as infliximab or tocilizumab, may represent more appropriate options. Emerging targeted therapies already used in iMG, including complement inhibitors and FcRN inhibitors, may also offer promising therapeutic perspectives.

ICIs are generally not used as first-line oncologic therapy and are currently discontinued in the presence of grade ≥ 3 adverse events. Improved risk stratification balancing oncologic benefit against neuromuscular toxicity may allow continuation of immunotherapy in selected patients as diagnostic and therapeutic strategies for triple-M syndrome improve.

## Data Availability

The original contributions presented in the study are included in the article/Supplementary material, further inquiries can be directed to the corresponding author.
