# Supplementary material for: Triple-M syndrome after immune checkpoint inhibitors: a systematic review of cases—early recognition is critical to survival
Source: Front Neurol. 2026 Aug 20;17:1861948. doi: 10.3389/fneur.2026.1861948 (PMC13537969; doi:10.3389/fneur.2026.1861948)
Supplement: Supplementary file 1 [file Table_1.docx]

**Supplementary Material**

Sommario

[Supplementary Table S1: CHECKLIST FOR CASE REPORTS	2](#_Toc237594702)

[Supplementary Table S2: CHECKLIST FOR CASE SERIE	4](#_Toc237594703)

[Supplementary Table S3 – Population clinical presentation	5](#_Toc237594704)

[Supplementary Table S4 – Serological Characteristics	8](#_Toc237594705)

[Supplementary Table S5– Diagnostic Workup and Treatment Initiation Strategy	11](#_Toc237594706)

[Supplementary Table S6 – Treatment Strategies and Clinical Outcomes	14](#_Toc237594707)

| Supplementary Table S1: CHECKLIST FOR CASE REPORTS | | | | | | | | | |
| --- | --- | --- | --- | --- | --- | --- | --- | --- | --- |
| Critical Appraisal tools for use in JBI Systematic Reviews | | | | | | | | | |
| **First author, year** | **Q1** | **Q2** | **Q3** | **Q4** | **Q5** | **Q6** | **Q7** | **Q8** | **Total** |
| Adeoye et al, 2024 (11) | Yes | Yes | Yes | Yes | Yes | Yes | Yes | Yes | 8/8 |
| Ahdi et al 2023 (13) | Yes | Unclear | Yes | Yes | Yes | Yes | Yes | Yes | 7/8 |
| Bai et al 2022 (15) | Yes | Yes | Yes | Yes | Yes | Yes | Yes | Yes | 8/8 |
| Baptista et al 2025 (16) | Yes | Yes | Yes | Yes | Yes | Yes | Yes | Yes | 8/8 |
| Basnet et al 2024 (17) | Yes | Yes | Yes | Yes | Yes | Yes | Yes | Yes | 8/8 |
| Bawek et al 2021 (18) | Yes | Yes | Yes | Yes | Yes | Yes | Yes | Yes | 8/8 |
| Cham et al 2021 (19) | Yes | Yes | Yes | Yes | Yes | Unclear | Yes | Yes | 7/8 |
| Chen et al 2021 (20) | Yes | Yes | Yes | Yes | Yes | Yes | Yes | Yes | 8/8 |
| Duong et al 2021 (21) | Yes | Unclear | Yes | Yes | Yes | Yes | Yes | Yes | 7/8 |
| Fazal et al 2020 (22) | Yes | Yes | Yes | Yes | Yes | Yes | Yes | Yes | 8/8 |
| Fazel et al 2019 (23) | Yes | Yes | Yes | Yes | Yes | Yes | Yes | Yes | 8/8 |
| Fuentes et al 2022 (24) | Yes | Yes | Yes | Yes | Yes | Yes | Yes | Yes | 8/8 |
| Giglio et al 2020 (25) | Yes | Yes | Yes | Yes | Yes | Yes | Yes | Yes | 8/8 |
| Hyun et al, 2020 (27) | Yes | Yes | Yes | Yes | Yes | Yes | Yes | Yes | 8/8 |
| Giovannini et al 2023 (26) | Yes | Yes | Yes | Yes | Yes | Yes | Yes | Yes | 8/8 |
| Jespersen 2021 (29) | Yes | Yes | Yes | Yes | Yes | Yes | Yes | Yes | 8/8 |
| Jeyakumar 2020 (30) | Yes | Yes | Yes | Yes | Yes | Yes | Yes | Yes | 8/8 |
| Kamikawa et al 2025 (31) | Yes | Yes | Yes | Yes | Yes | Yes | Yes | Yes | 8/8 |
| Karibea et al 2025 (32) | Yes | Yes | Yes | Yes | Yes | Yes | Yes | Yes | 8/8 |
| Kimura et al 2016 (33) | Yes | Yes | Yes | Yes | Yes | Yes | Yes | Yes | 8/8 |
| Komatsu et al 2021 (34) | Yes | Yes | Yes | Yes | Yes | Yes | Yes | Yes | 8/8 |
| Konstantina 2019 (35) | Yes | Yes | Yes | Yes | Yes | Yes | Yes | Yes | 8/8 |
| Lin et al. 2023 (36) | Yes | Yes | Yes | Yes | Yes | Yes | Yes | Yes | 8/8 |
| Luo et al 2021 (38) | Yes | Unclear | Yes | Yes | Yes | Yes | Yes | Yes | 7/8 |
| Mariniello et al 2024 (40) | Yes | Yes | Yes | Yes | Yes | Yes | Yes | Yes | 8/8 |
| Muranova et al 2023 (41) | Yes | Yes | Yes | Yes | Yes | Unclear | Yes | Yes | 7/8 |
| Nelke et al 2023 (42) | Yes | Yes | Yes | Yes | Yes | Yes | Yes | Yes | 8/8 |
| Newman et al 2024 (43) | Yes | Yes | Yes | Yes | Yes | Yes | Yes | Yes | 8/8 |
| Portolés Hernández et al 2021 (44) | Yes | Yes | Yes | Yes | Yes | Yes | Yes | Yes | 8/8 |
| Pourhassan et al 2019 (45) | Yes | Yes | Yes | Yes | Yes | Yes | Yes | Yes | 8/8 |
| Rhee et al 2022 (46) | Yes | Yes | Yes | Yes | Yes | Yes | Yes | Yes | 8/8 |
| Saishu et al 2022 (48) | Yes | Yes | Yes | Yes | Yes | Yes | Yes | Yes | 8/8 |
| Saito et al 2024 (49) | Yes | Yes | Yes | Yes | Yes | Yes | Yes | Yes | 8/8 |
| Sanchez-Sancho et al 2021 (51) | Yes | Yes | Yes | Yes | Yes | Yes | Unclear | Yes | 7/8 |
| Schiopu et al 2021 (52) | Yes | Yes | Yes | Yes | Yes | Yes | Yes | Yes | 8/8 |
| Schwab et al. 2022 (53) | Yes | Yes | Yes | Yes | Yes | Yes | Yes | Yes | 8/8 |
| Seker et al 2023 (54) | Yes | Yes | Yes | Yes | Yes | Yes | Yes | Yes | 8/8 |
| Shibuya et al 2024 (55) | Yes | Yes | Yes | Yes | Yes | Yes | Yes | Yes | 8/8 |
| Shirai et al 2019 (56) | Yes | Yes | Yes | Yes | Yes | Yes | Yes | Yes | 8/8 |
| So et al 2019 (57) | Yes | Yes | Yes | Yes | Yes | Yes | Yes | Yes | 8/8 |
| Takai et al2020 (58) | Yes | Yes | Yes | Yes | Yes | Yes | Yes | Yes | 8/8 |
| Todo et al 2020 (59) | Yes | Yes | Yes | Yes | Yes | Yes | Yes | Yes | 8/8 |
| Veccia et al 2020 (60) | Yes | Yes | Yes | Yes | Yes | Yes | Yes | Yes | 8/8 |
| Vermeulen et al 2020 (61) | Yes | Yes | Yes | Yes | Yes | Yes | Yes | Yes | 8/8 |
| Wang et al 2023 (62) | Yes | Yes | Yes | Yes | Yes | Yes | Yes | Yes | 8/8 |
| Wang et al 2022 (63) | Yes | Yes | Yes | Yes | Yes | Yes | Yes | Yes | 8/8 |
| Xie et al et al 2021 (65) | Yes | Yes | Yes | Yes | Yes | Yes | Yes | Yes | 8/8 |
| Xing et al 2020 (66) | Yes | Yes | Yes | Yes | Yes | Yes | Yes | Yes | 8/8 |
| Yanase et al 2021 (67) | Yes | Yes | Yes | Yes | Yes | Yes | Yes | Yes | 8/8 |
| Yin et al 2022 (68) | Yes | Yes | Yes | Yes | Yes | Yes | Yes | Yes | 8/8 |
| **Questions (Q):**  Q1: Were patient’s demographic characteristics clearly described?  Q2: Was the patient’s history clearly described and presented as a timeline?  Q3: Was the current clinical condition of the patient on presentation clearly described?  Q4: Were diagnostic tests or assessment methods and the results clearly described?  Q5: Was the intervention(s) or treatment procedure(s) clearly described?  Q6: Was the post-intervention clinical condition clearly described?  Q7: Were adverse events (harms) or unanticipated events identified and described?  Q8: Does the case report provide takeaway lessons?  Answers: Yes, No, Unclear or Not/Applicable | | | | | | | | | |

| Supplementary Table S2: CHECKLIST FOR CASE SERIE | | | | | | | | | | | |
| --- | --- | --- | --- | --- | --- | --- | --- | --- | --- | --- | --- |
| Critical Appraisal tools for use in JBI Systematic Reviews | | | | | | | | | | | |
| **First author, year** | **Q1** | **Q2** | **Q3** | **Q4** | **Q5** | **Q6** | **Q7** | **Q8** | **Q9** | **Q10** | **Total** |
| Aggarwal et al 2024 (12) | Yes | Yes | Yes | Unclear | Yes | Yes | Yes | Yes | No | No | 7/10 |
| Arora et al 2020 (14) | Unclear | Yes | Yes | Yes | Unclear | Yes | Yes | Yes | Yes | Yes | 8/10 |
| Hyun et al 2023 (28) | Yes | Yes | Yes | Yes | Yes | Yes | Yes | Yes | Yes | Yes | 10/10 |
| Jenkins et al 2022 (3) | No | Unclear | Unclear | No | Unclear | Yes | Yes | Yes | No | Not Applicable | 3/10 |
| Lipe et al 2021 (37) | Yes | Unclear | Yes | No | Unclear | Yes | Yes | Yes | No | Not Applicable | 5/10 |
| Marco et al 2022 (39) | Yes | Yes | Yes | Yes | Unclear | Yes | Yes | Yes | Yes | Not Applicable | 8/10 |
| Rossi et al 2023 (6) | Yes | Yes | Yes | Unclear | No | Yes | Yes | Yes | Yes | Yes | 8/10 |
| Rota et al 2018 (47) | No | Unclear | Unclear | No | No | Yes | Yes | Yes | No | Not Applicable | 3/10 |
| Sánchez-Camacho et al 2025 (50) | Yes | Yes | Yes | Unclear | Yes | Yes | Yes | Yes | Yes | Not Applicable | 8/10 |
| Weaver et al 2023 (64) | Yes | Yes | Yes | Unclear | Unclear | Yes | Yes | Yes | Yes | Not Applicable | 7/10 |
| Questions (Q):  Q1: Were there clear criteria for inclusion in the case series?  Q2: Was the condition measured in a standard, reliable way for all participants included in the case series?  Q3: Were valid methods used for identification of the condition for all participants included in the case series?  Q4: Did the case series have consecutive inclusion of participants?  Q5: Did the case series have complete inclusion of participants?  Q6: Was there clear reporting of the demographics of the participants in the study?  Q7: Was there clear reporting of clinical information of the participants?  Q8: Were the outcomes or follow up results of cases clearly reported?  Q9: Was there clear reporting of the presenting site(s)/clinic(s) demographic information?  Q10: Was statistical analysis appropriate?  Answers: Yes, No, Unclear or Not/Applicable | | | | | | | | | | | |

| Supplementary Table S3 – Population clinical presentation | | | | | | | | | | |
| --- | --- | --- | --- | --- | --- | --- | --- | --- | --- | --- |
| ***Authors*** | ***Sex*** | ***Age*** | ***Oncologic diagnosis*** | ***ICI*** | ***ICI-onset (days)*** | ***Ocular*** | ***Bulbar,***  ***dyspnea*** | ***Weakness,***  ***Myalgias*** | ***Cardiac*** | **EKG, Echocardio** |
| **Adeoye et al 2024 (11)** | **F** | **69** | **lung adenocarcinoma** | **Anti PDL1** | **21** | **No** | **YES** | **YES** | **No** | **cardiac electrical abnormality** |
| **Aggarwal et al. 2024 (12)** | **M** | **78** | **urothelial carcinoma** | **Anti PDL1** | **14** | **YES** | **No** | **YES** | **No** | **nn** |
|  | **M** | **86** | **melanoma** | **Anti PD1** | **14** | **YES** | **YES** | **No** | **No** | **cardiac electrical abnormality** |
| **Ahdi et al. 2023 (13)** | **M** | **58** | **renal cell carcinoma** | **Anti PD1** | **17,5** | **YES** | **No** | **YES** | **No** | **normal EF** |
| **Arora et al. 2020 (14)** | **M** | **70** | **melanoma** | **Combination** | **11** | **YES** | **No** | **No** | **YES** | **cardiac electrical abnormality, heart failure** |
|  | **M** | **79** | **melanoma** | **Anti PD1** | **26** | **YES** | **No** | **YES** | **No** | **cardiac electrical abnormality, normal EF** |
|  | **F** | **61** | **breast cancer** | **Combination** | **28** | **YES** | **YES** | **No** | **YES** | **cardiac electrical abnormality, normal EF** |
|  | **F** | **67** | **melanoma** | **Combination** | **14** | **No** | **YES** | **YES** | **No** | **normal EF** |
|  | **M** | **83** | **melanoma** | **Anti PD1** | **31** | **No** | **YES** | **YES** | **No** | **normal EF** |
|  | **M** | **70** | **renal cell carcinoma** | **Combination** | **21** | **No** | **YES** | **YES** | **No** | **normal EF** |
|  | **M** | **89** | **NSCLC** | **Anti PD1** | **32** | **YES** | **YES** | **No** | **No** | **cardiac electrical abnormality, normal EF** |
| **Bai et al. 2022 (15)** | **M** | **69** | **esophageal carcinoma** | **Anti PD1** | **21** | **YES** | **No** | **YES** | **YES** | **heart failure** |
| **Baptista et al. 2025 (16)** | **F** | **70** | **colon carcinoma** | **Anti PD1** | **14** | **YES** | **No** | **YES** | **No** | **nn** |
| **Basnet et al. 2024 (17)** | **M** | **76** | **melanoma** | **Combination** | **21** | **No** | **YES** | **No** | **YES** | **cardiac electrical abnormality, normal EF** |
| **Bawek et al. 2021 (18)** | **M** | **68** | **melanoma** | **Anti PD1** | **21** | **YES** | **YES** | **No** | **No** | **cardiac electrical abnormality, heart failure** |
| **Cham et al.2021 (19)** | **M** | **72** | **NSCLC** | **Anti PDL1** | **14** | **No** | **YES** | **YES** | **YES** | **ischemic changes, normal EF** |
| **Chen et al. 2021 (20)** | **M** | **77** | **chordoma** | **Anti PD1** | **21** | **YES** | **YES** | **No** | **YES** | **heart failure** |
| **Duong et al. 2021 (21)** | **F** | **57** | **melanoma** | **Combination** | **42** | **YES** | **No** | **YES** | **No** |  |
| **Fazal et al. 2020 (22)** | **M** | **82** | **melanoma** | **Anti PD1** | **31** | **No** | **YES** | **No** | **No** | **normal EF** |
| **Fazel et al. 2019 (23)** | **F** | **78** | **melanoma** | **Combination** | **5** | **YES** | **No** | **YES** | **No** |  |
| **Fuentes-Antrás et al. 2022 (24)** | **M** | **75** | **NSCLC** | **Anti PD1** | **21** | **No** | **No** | **YES** | **YES** | **cardiac electrical abnormality** |
| **Giglio et al. 2020 (25)** | **M** | **74** | **colon carcinoma** | **Anti PD1** | **29** | **No** | **YES** | **No** | **No** | **ischemic changes** |
| **Giovannini et al 2023 (26)** | **M** | **65** | **melanoma** | **Anti PD1** | **12** | **No** | **YES** | **YES** | **No** | **cardiac electrical abnormality** |
| **Hyun et al. 2020 (27)** | **F** | **45** | **thymoma** | **Anti PD1** | **12** | **No** | **YES** | **YES** | **No** | **cardiac electrical abnormality, heart failure** |
| **Hyun et al. 2023 (28)** | **F** | **55** | **thymoma** | **Anti PD1** | **15** | **YES** | **YES** | **YES** | **No** |  |
|  | **F** | **64** | **thymoma** | **Anti PD1** | **12** | **YES** | **YES** | **YES** | **No** |  |
| **Jenkins et al. 2022 (3)** | **M** | **71** | **melanoma** | **Anti PD1** | **47** | **YES** | **YES** | **No** | **No** | **cardiac electrical abnormality, normal EF** |
|  | **F** | **65** | **SCLC** | **Anti PD1** | **50** | **YES** | **YES** | **No** | **No** | **normal EF** |
| **Jespersen et al. 2021 (29)** | **M** | **57** | **renal cell carcinoma** | **Combination** | **12** | **YES** | **No** | **YES** | **YES** | **cardiac electrical abnormality, heart failure** |
| **Jeyakumar et al.2020 (30)** | **M** | **86** | **squamous cell carcinoma** | **anti PD1** | **16** | **No** | **No** | **YES** | **No** | **cardiac electrical abnormality** |
| **Kamikawa et al.2025 (31)** | **F** | **80** | **urothelial carcinoma** | **Anti PD1** | **18** | **YES** | **No** | **No** | **No** | **cardiac electrical abnormality, heart failure** |
| **Karibea et al. 2025 (32)** | **F** | **76** | **renal cell carcinoma** | **Anti PDL1** | **23** | **YES** | **YES** | **YES** | **No** | **normal EF** |
| **Kimura et al. 2016 (33)** | **M** | **80** | **melanoma** | **Anti PD1** | **14** | **No** | **YES** | **YES** | **No** | **cardiac electrical abnormality** |
| **Komatsu et al. 2021 (34)** | **M** | **66** | **gastric cancer** | **Anti PD1** | **24** | **YES** | **YES** | **YES** | **No** | **cardiac electrical abnormality** |
| **Konstantina et al. 2019 (35)** | **F** | **30** | **thymoma** | **Anti PD1** | **3** | **YES** | **YES** | **YES** | **YES** | **ischemic changes** |
| **Lin et al 2023 (36)** | **M** | **51** | **SCLC** | **Anti PD1** | **19** | **No** | **YES** | **YES** | **YES** | **cardiac electrical abnormality** |
| **Lipe et al. 2021 (37)** | **F** | **49** | **thymoma** | **Anti PD1** | **21** | **No** | **YES** | **YES** | **No** |  |
|  | **M** | **67** | **SCLC** | **anti PDL1** | **28** | **No** | **No** | **YES** | **No** |  |
|  | **M** | **70** | **urothelial carcinoma** | **Anti PD1** | **25** | **YES** | **YES** | **YES** | **No** |  |
|  | **F** | **81** | **renal cell carcinoma** | **Combination** | **92** | **No** | **YES** | **No** | **No** |  |
|  | **M** | **75** | **chordoma** | **Anti PD1** | **62** | **YES** | **No** | **No** | **No** |  |
|  | **F** | **66** | **renal cell carcinoma** | **Combination** | **132** | **No** | **YES** | **YES** | **No** |  |
|  | **F** | **74** | **melanoma** | **Combination** | **30** | **YES** | **YES** | **No** | **No** |  |
| **Luo et al. 2021 (38)** | **F** | **47** | **thymoma** | **anti PD1** | **21** | **YES** | **No** | **YES** | **No** | **cardiac electrical abnormality** |
| **Marco et al. 2022 (39)** | **M** | **77** | **NSCLC** | **Anti PD1** | **60** | **YES** | **YES** | **YES** | **YES** |  |
|  | **M** | **78** | **prostate carcinoma** | **Anti PD1** | **19** | **YES** | **YES** | **No** | **No** |  |
|  | **M** | **70** | **melanoma** | **Anti PD1** | **15** | **No** | **YES** | **YES** | **No** |  |
|  | **M** | **85** | **NSCLC** | **anti PDL1** | **37** | **YES** | **YES** | **YES** | **No** |  |
| **Mariniello et al. 2024 (40)** | **F** | **40** | **lung adenocarcinoma** | **Combination** | **30** | **YES** | **YES** | **YES** | **YES** | **cardiac electrical abnormality, ischemic changes, echocardio inflammatory** |
| **Muranova et al. 2023 (41)** | **F** | **34** | **thymoma** | **Anti PD1** | **5** | **YES** | **YES** | **YES** | **No** | **ischemic changes** |
| **Nelke et al. 2023 (42)** | **F** | **47** | **breast cancer** | **Anti PD1** | **15** | **No** | **YES** | **YES** | **No** |  |
| **Newman et al. 2024 (43)** | **M** | **87** | **urothelial carcinoma** | **Anti PD1** | **21** | **YES** | **No** | **YES** | **No** | **echocardio hyperdynamic** |
| **Portolés Hernández et al. 2021 (44)** | **F** | **48** | **thymoma** | **Anti PD1** | **10** | **YES** | **YES** | **No** | **No** | **cardiac electrical abnormality, heart failure** |
| **Pourhassan et al. 2019 (45)** | **M** | **85** | **melanoma** | **Anti PD1** | **15** | **No** | **YES** | **YES** | **No** | **(previous AF), ischemic changes** |
| **Rhee et al. 2022 (46)** | **M** | **73** | **melanoma** | **Anti PD1** | **30** | **YES** | **YES** | **YES** | **No** | **heart failure** |
| **Rossi et al. 2023 (6)** |  | **73,5** | **melanoma** | **Anti PD1** | **14** | **YES** | **YES** | **YES** | **No** |  |
|  |  | **73,5** | **melanoma** | **Anti PD1** | **35** | **YES** | **YES** | **YES** | **No** |  |
|  |  | **73,5** | **melanoma** | **Combination** | **56** | **YES** | **YES** | **YES** | **No** |  |
| **Rota et al. 2018 (47)** | **F** | **72** | **renal cell carcinoma** | **Anti PD1** | **14** | **No** | **No** | **YES** | **No** | **cardiac electrical abnormality** |
|  | **M** | **71** | **renal cell carcinoma** | **Anti PD1** | **14** | **No** | **No** | **YES** | **No** |  |
| **Saishu et al. 2022 (48)** | **F** | **55** | **melanoma** | **Anti PD1** | **14** | **YES** | **YES** | **YES** | **No** | **cardiac electrical abnormality** |
| **Saito et al. 2024 (49)** | **M** | **78** | **NSCLC** | **Anti PDL1** | **25** | **No** | **YES** | **YES** | **YES** | **ischemic changes, heart failure** |
| **Sánchez-Camacho et al. 2025 (50)** | **M** | **75** | **melanoma** | **Combination** | **15** | **YES** | **No** | **No** | **YES** | **cardiac electrical abnormality** |
|  | **F** | **42** | **melanoma** | **Anti PD1** | **30** | **YES** | **No** | **YES** | **No** |  |
|  | **F** | **59** | **renal cell carcinoma** | **Anti PD1** | **35** | **YES** | **YES** | **YES** | **No** | **ischemic changes, normal EF** |
|  | **M** | **70** | **gastric cancer** | **Anti PD1** | **21** | **No** | **No** | **YES** | **No** | **cardiac electrical abnormality, normal EF** |
| **Sanchez-Sancho et al. 2021 (51)** | **M** | **63** | **liposarcoma** | **Anti PD1** | **6** | **YES** | **YES** | **YES** | **No** | **cardiac electrical abnormality, heart failure** |
| **Schiopu et al. 2021 (52)** | **M** | **75** | **mesothelioma** | **Anti PD1** | **21** | **YES** | **YES** | **YES** | **No** | **cardiac electrical abnormality, ischemic changes, heart failure** |
| **Schwab et al 2022 (53)** | **M** | **81** | **urothelial carcinoma** | **Anti PD1** | **42** | **No** | **No** | **YES** | **No** | **nn** |
| **Seker et al. 2023 (54)** | **F** | **31** | **thymoma** | **Anti PD1** | **18** | **YES** | **YES** | **YES** | **No** | **cardiac electrical abnormality, normal EF** |
| **Shibuya et al. 2024 (55)** | **M** | **83** | **HCC** | **Anti PDL1** | **28** | **YES** | **No** | **YES** | **No** | **cardiac electrical abnormality, ischemic changes, normal EF** |
| **Shirai et al. 2019 (56)** | **M** | **83** | **melanoma** | **Anti PD1** | **25** | **YES** | **No** | **YES** | **No** | **cardiac electrical abnormality, normal EF** |
| **So et al. 2019 (57)** | **F** | **55** | **melanoma** | **Anti PD1** | **15** | **YES** | **YES** | **YES** | **No** | **cardiac electrical abnormality, heart failure** |
| **Takai et al. 2020 (58)** | **M** | **77** | **urothelial carcinoma** | **Anti PD1** | **20** | **YES** | **No** | **No** | **No** | **cardiac electrical abnormality, heart failure** |
| **Todo et al. 2020 (59)** | **M** | **63** | **urothelial carcinoma** | **Anti PD1** | **12** | **YES** | **No** | **No** | **No** | **cardiac electrical abnormality, normal EF** |
| **Veccia et al. 2020 (60)** | **M** | **65** | **SCLC** | **Anti PD1** | **27** | **YES** | **No** | **YES** | **No** | **cardiac electrical abnormality, heart failure** |
| **Vermeulen et al. 2020 (61)** | **F** | **53** | **melanoma** | **Anti CTLA4** | **21** | **YES** | **YES** | **No** | **No** | **ischemic changes** |
| **Wang et al. 2023 (62)** | **F** | **45** | **thymoma** | **Anti PD1** | **14** | **YES** | **No** | **No** | **YES** | **cardiac electrical abnormality, heart failure** |
| **Wang et al. 2022 (63)** | **M** | **65** | **colon carcinoma** | **Anti PD1** | **20** | **YES** | **No** | **YES** | **No** |  |
| **Weaver et al. 2023 (64)** | **M** | **56** | **renal cell carcinoma** | **Combination** | **14** | **YES** | **No** | **No** | **No** |  |
|  | **M** | **45** | **melanoma** | **Combination** | **28** | **YES** | **No** | **No** | **No** | **normal EF** |
|  | **M** | **58** | **melanoma** | **Combination** | **14** | **YES** | **YES** | **No** | **No** | **cardiac electrical abnormality, heart failure, normal EF** |
|  | **M** | **73** | **melanoma** | **Combination** | **14** | **YES** | **No** | **YES** | **No** | **cardiac electrical abnormality, normal EF** |
|  | **M** | **76** | **melanoma** | **Anti PD1** | **42** | **YES** | **YES** | **YES** | **No** | **normal EF** |
|  | **M** | **80** | **melanoma** | **Anti PD1** | **42** | **YES** | **No** | **No** | **No** | **heart failure** |
|  | **M** | **75** | **renal cell carcinoma** | **Anti PD1** | **28** | **YES** | **No** | **No** | **No** | **heart failure** |
|  | **F** | **82** | **colon carcinoma** | **Anti PD1** | **42** | **YES** | **No** | **YES** | **No** | **normal EF** |
|  | **M** | **86** | **melanoma** | **Anti PD1** | **28** | **YES** | **No** | **YES** | **No** | **normal EF** |
|  | **M** | **76** | **urothelial carcinoma** | **Anti PDL1** | **14** | **YES** | **No** | **No** | **No** |  |
| **Xie et al. 2021 (65)** | **M** | **67** | **lung NET** | **Anti PD1** | **14** | **YES** | **YES** | **YES** | **No** | **cardiac electrical abnormality, normal EF** |
| **Xing et al. 2020 (66)** | **M** | **66** | **NSCLC** | **Anti PD1** | **25** | **No** | **YES** | **YES** | **No** | **cardiac electrical abnormality, normal EF** |
| **Yanase et al. 2020 (67)** | **M** | **59** | **renal cell carcinoma** | **Combination** | **21** | **YES** | **No** | **No** | **No** | **cardiac electrical abnormality** |
| **Yin et al. 2022 (68)** | **F** | **71** | **cholangiocarcinoma** | **Anti PD1** | **14** | **YES** | **YES** | **YES** | **No** | **cardiac electrical abnormality, normal EF** |

AF = Atrial fibrillation; Anti-CTLA4 = Anti–cytotoxic T-lymphocyte antigen-4 therapy; Anti-PD1 = Anti–programmed cell death protein 1 therapy; Anti-PDL1 = Anti–programmed death-ligand 1 therapy; Combination = Combined immunotherapy; Echocardio = Echocardiography; EF = Ejection fraction; EKG = Electrocardiogram; F = Female; HCC = Hepatocellular carcinoma; ICI = Immune checkpoint inhibitors; M = Male; NET = Neuroendocrine tumor; NSCLC = Non–small cell lung cancer; SCLC = Small cell lung cancer; nn = not reported / no data available

| Supplementary Table S4 – Serological Characteristics | | | | | |
| --- | --- | --- | --- | --- | --- |
| ***Authors*** | **MG autoimmunity** | **Anti-Striate Muscle Ab** | **CPK elevation** | **U/L** | **Troponin elevation** |
| **Adeoye et al 2024 (11)** | **neg** | **nd** | **Yes** | **8668** | **TnT** |
| **Aggarwal et al. 2024 (12)** | **AChR+** | **nd** | **Yes** | **8500** | **TnT, TnI** |
|  | **AChR+** | **nd** | **Yes** | **6185** | **TnT** |
| **Ahdi et al. 2023 (13)** | **neg** | **neg** | **Yes** | **11205** | **Yes** |
| **Arora et al. 2020 (14)** | **AChR+** | **nd** | **Yes** | **3549** | **Yes** |
|  | **neg** | **nd** | **Yes** | **11953** | **Yes** |
|  | **neg** | **nd** | **Yes** | **8082** | **Yes** |
|  | **AChR+** | **nd** | **Yes** | **7244** | **Yes** |
|  | **neg** | **nd** | **Yes** | **2886** | **Unspecified** |
|  | **neg** | **nd** | **Yes** | **11000** | **Yes** |
|  | **neg** | **nd** | **Yes** | **3843** | **Yes** |
| **Bai et al. 2022 (15)** | **AChR+** | **nd** | **Yes** | **3503** | **TnI** |
| **Baptista et al. 2025 (16)** | **neg** | **nd** | **Yes** | **37510** | **TnI** |
| **Basnet et al. 2024 (17)** | **AChR+** | **nd** | **Yes** | **6235** | **TnI** |
| **Bawek et al. 2021 (18)** | **neg** | **neg** | **Yes** | **nd** | **Yes** |
| **Cham et al.2021 (19)** | **AChR+** | **nd** | **Yes** | **9262** | **Yes** |
| **Chen et al. 2021 (20)** | **AChR+** | **pos** | **Yes** | **706** | **TnI** |
| **Duong et al. 2021 (21)** | **AChR+** | **nd** | **Yes** | **9000** | **Yes** |
| **Fazal et al. 2020 (22)** | **nd** | **nd** | **Yes** | **3658** | **TnI** |
| **Fazel et al. 2019 (23)** | **nd** | **pos** | **Yes** | **9198** | **TnI** |
| **Fuentes-Antrás et al. 2022 (24)** | **nd** | **pos** | **Yes** | **5300** | **Yes** |
| **Giglio et al. 2020 (25)** | **AChR+** | **pos** | **Yes** | **1276** | **TnT** |
| **Giovannini et al 2023 (26)** | **nd** | **nd** | **Yes** | **14977** | **TnI** |
| **Hyun et al. 2020 (27)** | **AChR+ (previously known)** | **nd** | **Yes** | **7552** | **TnI** |
| **Hyun et al. 2023 (28)** | **AChR+** | **nd** | **Yes** | **7552** | **Unspecified** |
|  | **AChR+** | **nd** | **Yes** | **1916** | **Unspecified** |
| **Jenkins et al. 2022 (3)** | **neg** | **nd** | **Yes** | **1604** | **Yes** |
|  | **neg** | **nd** | **Yes** | **2993** | **Yes** |
| **Jespersen et al. 2021 (29)** | **AChR+** | **pos** | **Yes** | **3500** | **Yes** |
| **Jeyakumar et al.2020 (30)** | **AChR+** | **nd** | **Yes** | **6407** | **TnT, TnI** |
| **Kamikawa et al.2025 (31)** | **AChR+** | **nd** | **Yes** | **6417** | **TnT** |
| **Karibea et al. 2025 (32)** | **AChR+** | **pos** | **Yes** | **7801** | **TnI** |
| **Kimura et al. 2016 (33)** | **AChR+** | **nd** | **Yes** | **7740** | **Unspecified** |
| **Komatsu et al. 2021 (34)** | **AChR+** | **pos** | **Yes** | **8903** | **TnI** |
| **Konstantina et al. 2019 (35)** | **AChR+** | **nd** | **Yes** | **nd** | **Yes** |
| **Lin et al 2023 (36)** | **nd** | **pos** | **Yes** | **nd** | **TnI** |
| **Lipe et al. 2021 (37)** | **nd** | **pos** | **Yes** | **13605** | **TnT** |
|  | **nd** | **nd** | **Yes** | **5486** | **TnT** |
|  | **nd** | **nd** | **Yes** | **12706** | **TnT** |
|  | **nd** | **nd** | **Yes** | **236** | **TnT** |
|  | **nd** | **pos** | **Yes** | **1956** | **TnT** |
|  | **nd** | **nd** | **Yes** | **61223** | **TnT** |
|  | **nd** | **pos** | **Yes** | **747** | **TnT** |
| **Luo et al. 2021 (38)** | **AChR+** | **pos** | **Yes** | **25200** | **TnI** |
| **Marco et al. 2022 (39)** | **AChR+** | **nd** | **Yes** | **1680** | **TnT** |
|  | **AChR+** | **nd** | **Yes** | **1120** | **TnT** |
|  | **AChR+** | **nd** | **Yes** | **2520** | **TnT** |
|  | **AChR+** | **nd** | **Unspecified** |  | **TnT** |
| **Mariniello et al. 2024 (40)** | **neg** | **nd** | **Yes** | **6388** | **TnI** |
| **Muranova et al. 2023 (41)** | **AChR+** | **nd** | **Yes** | **3621** | **TnI** |
| **Nelke et al. 2023 (42)** | **AChR+ (previously known)** | **nd** | **Yes** | **3300** | **Yes** |
| **Newman et al. 2024 (43)** | **neg** | **pos** | **Yes** | **6042** | **Yes** |
| **Portolés Hernández et al. 2021 (44)** | **AChR+** | **nd** | **Yes** | **2430** | **TnI** |
| **Pourhassan et al. 2019 (45)** | **nd** | **nd** | **Yes** | **7000** | **Yes** |
| **Rhee et al. 2022 (46)** | **AChR+** | **nd** | **Yes** | **4986** | **TnT** |
| **Rossi et al. 2023 (6)** | **AChR+** | **nd** | **Yes** | **6551** | **Unspecified** |
|  | **AChR+** | **nd** | **Yes** | **6551** | **Unspecified** |
|  | **AChR+** | **nd** | **Yes** | **6551** | **Unspecified** |
| **Rota et al. 2018 (47)** | **nd** | **nd** | **Unspecified** |  | **Unspecified** |
|  | **nd** | **nd** | **Yes** |  | **Unspecified** |
| **Saishu et al. 2022 (48)** | **AChR+ (previously known)** | **nd** | **Yes** | **13603** | **Unspecified** |
| **Saito et al. 2024 (49)** | **neg** | **pos** | **Yes** | **9385** | **TnT** |
| **Sánchez-Camacho et al. 2025 (50)** | **neg** | **pos** | **Yes** | **2187** | **TnI** |
|  | **neg** | **neg** | **Yes** | **2021** | **TnI** |
|  | **neg** | **pos** | **Yes** | **4157** | **TnI** |
|  | **neg** | **neg** | **Yes** | **485** | **TnT** |
| **Sanchez-Sancho et al. 2021 (51)** | **AChR+** | **nd** | **Yes** | **14448** | **Yes** |
| **Schiopu et al. 2021 (52)** | **neg** | **pos** | **Yes** | **3480** | **TnT** |
| **Schwab et al 2022 (53)** | **neg** | **nd** | **Yes** | **9244** | **Yes** |
| **Seker et al. 2023 (54)** | **AChR+ (previously known)** | **nd** | **Yes** | **10296** | **TnT** |
| **Shibuya et al. 2024 (55)** | **AChR+** | **pos** | **Yes** | **7672** | **TnI** |
| **Shirai et al. 2019 (56)** | **AChR+** | **nd** | **Yes** | **5567** | **TnT** |
| **So et al. 2019 (57)** | **AChR+ (previously known)** | **nd** | **Yes** | **13652** | **Yes** |
| **Takai et al. 2020 (58)** | **AChR+** | **nd** | **Yes** | **8574** | **TnT** |
| **Todo et al. 2020 (59)** | **neg** | **nd** | **Yes** | **3385** | **TnI** |
| **Veccia et al. 2020 (60)** | **neg** | **nd** | **Yes** | **3844** | **TnT** |
| **Vermeulen et al. 2020 (61)** | **neg** | **nd** | **Yes** | **2537** | **TnT** |
| **Wang et al. 2023 (62)** | **AChR+** | **nd** | **Yes** | **22536** | **TnI** |
| **Wang et al. 2022 (63)** | **AChR+** | **nd** | **Yes** | **11920** | **TnI** |
| **Weaver et al. 2023 (64)** | **AChR+** | **nd** | **Yes** | **3763** | **Unspecified** |
|  | **nd** | **nd** | **Unspecified** |  | **Yes** |
|  | **MuSK+** | **nd** | **Yes** | **2922** | **Yes** |
|  | **AChR+** | **nd** | **Yes** | **5881** | **Yes** |
|  | **AChR+** | **nd** | **Yes** | **4140** | **Yes** |
|  | **nd** | **nd** | **Yes** | **985** | **Yes** |
|  | **neg** | **nd** | **Yes** | **1214** | **Yes** |
|  | **AChR+** | **nd** | **Yes** | **1528** | **Yes** |
|  | **AChR+** | **nd** | **Yes** | **405** | **Yes** |
|  | **AChR+** | **nd** | **Yes** | **1249** | **Unspecified** |
| **Xie et al. 2021 (65)** | **nd** | **nd** | **Yes** | **4256** | **TnI** |
| **Xing et al. 2020 (66)** | **AChR+ (previously known)** | **nd** | **Yes** | **11919** | **TnT** |
| **Yanase et al. 2020 (67)** | **AChR+** | **nd** | **Yes** | **8944** | **TnI** |
| **Yin et al. 2022 (68)** | **AChR+** | **nd** | **Yes** | **1658** | **Yes** |

AChR+ = Acetylcholine receptor antibody positive; MuSK+ = Muscle-specific kinase antibody positive; Anti-striate muscle Ab = Anti–striated muscle antibodies; CPK = Creatine phosphokinase (creatine kinase); MG = Myasthenia gravis; neg = negative; nd = not determined / no data available; pos = positive; TnI = Troponin I; TnT = Troponin T

| Supplementary Table S5– Diagnostic Workup and Treatment Initiation Strategy | | | | | |  |
| --- | --- | --- | --- | --- | --- | --- |
| ***Authors*** | **Biopsy** | **Cardiac MRI** | **Muscle MRI** | **EMG** | **pattern EMG** | **Empiric therapy versus First the exams** |
| **Adeoye et al 2024 (11)** | **No** | **No** | **YES** | **YES** | **myo, NMJ-** | **first the exams** |
| **Aggarwal et al. 2024 (12)** | **No** | **No** | **No** | **No** |  |  |
|  | **No** | **No** | **No** | **No** |  |  |
| **Ahdi et al. 2023 (13)** | **No** | **No** | **No** | **YES** | **myo** | **empiric therapy** |
| **Arora et al. 2020 (14)** | **No** | **YES** | **No** | **No** |  | **empiric therapy** |
|  | **No** | **No** | **No** | **No** |  |  |
|  | **No** | **No** | **No** | **No** |  |  |
|  | **No** | **YES** | **No** | **No** |  | **empiric therapy** |
|  | **No** | **No** | **No** | **No** |  |  |
|  | **No** | **No** | **No** | **No** |  |  |
|  | **No** | **YES** | **No** | **YES** | **myo** | **empiric therapy** |
| **Bai et al. 2022 (15)** | **No** | **YES** | **No** | **YES** | **myo, deltoid** | **first EMG, post MRI** |
| **Baptista et al. 2025 (16)** | **No** | **YES** | **No** | **YES** | **myo** | **first MRI, post EMG** |
| **Basnet et al. 2024 (17)** | **No** | **No** | **No** | **No** |  |  |
| **Bawek et al. 2021 (18)** | **No** | **No** | **No** | **No** | **ice pack test +** |  |
| **Cham et al.2021 (19)** | **No** | **No** | **No** | **No** |  |  |
| **Chen et al. 2021 (20)** | **No** | **YES** | **No** | **No** |  | **first the exams** |
| **Duong et al. 2021 (21)** | **No** | **No** | **No** | **No** |  |  |
| **Fazal et al. 2020 (22)** | **No** | **No** | **No** | **No** |  |  |
| **Fazel et al. 2019 (23)** | **No** | **No** | **YES** | **No** |  | **empiric therapy** |
| **Fuentes-Antrás et al. 2022 (24)** | **autopsy** | **No** | **No** | **No** |  |  |
| **Giglio et al. 2020 (25)** | **autopsy** | **No** | **No** | **No** |  |  |
| **Giovannini et al 2023 (26)** | **autopsy** | **No** | **No** | **No** |  |  |
| **Hyun et al. 2020 (27)** | **No** | **No** | **No** | **No** |  |  |
| **Hyun et al. 2023 (28)** | **No** | **No** | **No** | **No** |  |  |
|  | **No** | **No** | **No** | **YES** | **NMJ +** | **nd** |
| **Jenkins et al. 2022 (3)** | **muscle** | **YES** | **No** | **YES** | **myo, NMJ -** | **first the exams** |
|  | **No** | **YES** | **No** | **YES** | **myo, NMJ -** | **first the exams** |
| **Jespersen et al. 2021 (29)** | **No** | **No** | **No** | **YES** | **myo, NMJ -** | **empiric therapy** |
| **Jeyakumar et al.2020 (30)** | **cardiac** | **No** | **No** | **No** |  | **empiric therapy** |
| **Kamikawa et al.2025 (31)** | **cardiac** | **No** | **No** | **No** |  | **empiric therapy** |
| **Karibea et al. 2025 (32)** | **No** | **YES** | **No** | **YES** | **myo, NMJ -** | **first the exams** |
| **Kimura et al. 2016 (33)** | **muscle** | **No** | **No** | **No** |  | **first the exams** |
| **Komatsu et al. 2021 (34)** | **cardiac** | **No** | **No** | **No** |  | **first the exams** |
| **Konstantina et al. 2019 (35)** | **No** | **No** | **No** | **No** |  |  |
| **Lin et al 2023 (36)** | **muscle** | **No** | **No** | **YES** | **myo** | **first the exams** |
| **Lipe et al. 2021 (37)** | **cardiac** | **No** | **No** | **No** |  | **nd** |
|  | **No** | **No** | **No** | **No** |  |  |
|  | **No** | **No** | **No** | **No** |  |  |
|  | **cardiac** | **No** | **No** | **No** |  | **nd** |
|  | **No** | **No** | **No** | **No** |  |  |
|  | **cardiac/muscle** | **No** | **No** | **No** |  | **nd** |
|  | **No** | **No** | **No** | **No** |  |  |
| **Luo et al. 2021 (38)** | **No** | **No** | **No** | **YES** | **myo, NMJ - (biceps brachii, extensor digitorum communis, quadriceps femoris)** | **first the exams** |
| **Marco et al. 2022 (39)** | **No** | **No** | **No** | **YES** | **myo** | **nd** |
|  | **No** | **No** | **No** | **YES** | **myo** | **nd** |
|  | **No** | **No** | **No** | **YES** | **myo, NMJ +** | **nd** |
|  | **No** | **No** | **No** | **YES** | **myo, NMJ +** | **nd** |
| **Mariniello et al. 2024 (40)** | **muscle** | **YES** | **No** | **YES** | **NMJ-** | **first the exams** |
| **Muranova et al. 2023 (41)** | **No** | **No** | **No** | **No** |  |  |
| **Nelke et al. 2023 (43)** | **muscle** | **YES** | **YES** | **YES** | **myo, gastrocnemius** | **first EMG, post MRIs and biopsy** |
| **Newman et al. 2024 (42)** | **No** | **No** | **YES** | **No** |  | **first the exams** |
| **Portolés Hernández et al. 2021 (44)** | **cardiac** | **No** | **No** | **No** |  | **first the exams** |
| **Pourhassan et al. 2019 (45)** | **No** | **No** | **No** | **No** |  |  |
| **Rhee et al. 2022 (46)** | **No** | **No** | **No** | **YES** | **myo (deltoid), NMJ +** | **nd** |
| **Rossi et al. 2023 (6)** | **no dati grezzi** | **No** | **No** | **No** |  | **nd** |
|  | **no dati grezzi** | **No** | **No** | **No** |  | **nd** |
|  | **no dati grezzi** | **No** | **No** | **No** |  | **nd** |
| **Rota et al. 2018 (47)** | **No** | **No** | **No** | **YES** | **NMJ +** | **first the exams** |
|  | **No** | **No** | **No** | **YES** | **NMJ +** | **first the exams** |
| **Saishu et al. 2022 (48)** | **No** | **No** | **YES** | **No** |  | **empiric therapy** |
| **Saito et al. 2024 (49)** | **No** | **No** | **No** | **YES** | **myo (trapezius), NMJ+** | **empiric therapy** |
| **Sánchez-Camacho et al. 2025 (50)** | **No** | **No** | **No** | **YES** | **myo, NMJ-** | **empiric therapy** |
|  | **No** | **No** | **No** | **YES** | **myo, NMJ-** | **empiric therapy** |
|  | **No** | **YES** | **No** | **No** |  | **empiric therapy** |
|  | **No** | **YES** | **No** | **YES** | **myo (proximal), NMJ-** | **empiric therapy** |
| **Sanchez-Sancho et al. 2021 (51)** | **muscle** | **No** | **No** | **YES** | **myo (proximal), NMJ -** | **nd** |
| **Schiopu et al. 2021 (52)** | **No** | **No** | **No** | **No** |  |  |
| **Schwab et al 2022 (53)** | **muscle (thigh)** | **No** | **YES** | **YES** | **NMJ +** | **empiric therapy** |
| **Seker et al. 2023 (54)** | **No** | **No** | **No** | **YES** | **myo (tibialis anterior, gastrocnemius, rectus femoris, deltoid, biceps, forearm flexor, and extensor)** | **first the exams** |
| **Shibuya et al. 2024 (55)** | **cardiac** | **No** | **No** | **YES** | **NMJ-** | **first the exams** |
| **Shirai et al. 2019 (56)** | **No** | **No** | **YES** | **YES** | **NMJ -** | **first the exams** |
| **So et al. 2019 (57)** | **muscle** | **No** | **YES** | **YES** | **NMJ +** | **first the exams** |
| **Takai et al. 2020 (58)** | **No** | **No** | **No** | **No** |  |  |
| **Todo et al. 2020 (59)** | **No** | **No** | **No** | **YES** | **EMG normal, NMJ -** | **first the exams** |
| **Veccia et al. 2020 (60)** | **No** | **No** | **No** | **YES** | **myo (proximal)** | **first the exams** |
| **Vermeulen et al. 2020 (61)** | **muscle** | **No** | **No** | **YES** | **myo, NMJ -** | **first the exams** |
| **Wang et al. 2023 (62)** | **No** | **No** | **No** | **YES** | **NMJ borderline** | **first the exams** |
| **Wang et al. 2022 (63)** | **No** | **No** | **No** | **YES** | **normal (neostigmine test +)** | **first the exams** |
| **Weaver et al. 2023 (64)** | **No** | **No** | **No** | **No** |  |  |
|  | **No** | **No** | **No** | **No** |  |  |
|  | **No** | **No** | **No** | **No** |  |  |
|  | **No** | **No** | **No** | **No** |  |  |
|  | **No** | **No** | **No** | **No** |  |  |
|  | **No** | **No** | **No** | **No** |  |  |
|  | **No** | **No** | **No** | **No** |  |  |
|  | **No** | **No** | **No** | **No** |  |  |
|  | **No** | **No** | **No** | **No** |  |  |
|  | **No** | **No** | **No** | **No** |  |  |
| **Xie et al. 2021 (65)** | **muscle** | **No** | **No** | **No** |  | **empiric therapy** |
| **Xing et al. 2020 (66)** | **No** | **No** | **No** | **No** |  |  |
| **Yanase et al. 2020 (67)** | **No** | **No** | **No** | **No** |  |  |
| **Yin et al. 2022 (68)** | **No** | **No** | **No** | **YES** | **normal** | **first EMG, post MRI** |

EMG = Electromyography; MRI = Magnetic Resonance Imaging; NMJ = Neuromuscular junction; nd = not determined / no data available; NMJ+ = positive neuromuscular junction involvement; NMJ− = negative neuromuscular junction involvement; myo = myopathic pattern (myopathy).

| Supplementary Table S6 – Treatment Strategies and Clinical Outcomes | | | | | | | | |
| --- | --- | --- | --- | --- | --- | --- | --- | --- |
| ***Authors*** | **STEROIDS** | **IVIg/**  **PEX/**  **thymoglob** | **IMMUNO**  **SUPPRESSANTS** | **AB MONOCLONAL** | **SYMPTOMATIC TREATMENT** | **therapy mode** | **Prognosis** | **Complications** |
| **Adeoye et al 2024 (11)** | **Yes 1** | **Yes 2** | **No** | **No** | **No** | **escalation** | **recover with successful discharge** |  |
| **Aggarwal et al. 2024 (12)** | **Yes 1** | **Yes 2** | **Yes 3** | **No** | **Pyridostigmine** | **escalation** | **recover with successful discharge** |  |
|  | **Yes 1** | **Yes 2** | **No** | **No** | **Neostigmine** | **escalation** | **in-hospital death** | **respiratory failure** |
| **Ahdi et al. 2023 (13)** | **Yes 1** | **Yes 2** | **No** | **No** | **No** | **combination** | **recover with successful discharge** | **ir thrombocytopenia** |
| **Arora et al. 2020 (14)** | **Yes 1** | **Yes 2,5** | **Yes 3,4** | **No** | **No** | **escalation** | **in-hospital death** | **respiratory failure** |
|  | **Yes 1** | **Yes 2,5** | **Yes 3,4** | **No** | **No** | **escalation** | **in-hospital death** |  |
|  | **Yes 1** | **No** | **Yes 2** | **No** | **Pyridostigmine** | **escalation** | **in-hospital death** | **SIADH, respiratory failure** |
|  | **Yes 1** | **Yes 3,4** | **Yes 2** | **No** | **No** | **combination** | **in-hospital death** |  |
|  | **Yes 1** | **Yes 2** | **No** | **No** | **No** | **combination** | **in-hospital death** | **respiratory failure** |
|  | **Yes 1** | **Yes 2** | **No** | **No** | **No** | **escalation** | **in-hospital death** | **upper GI bleeding, respiratory failure** |
|  | **Yes 1** | **No** | **No** | **No** | **No** | **mono** | **in-hospital death** |  |
| **Bai et al. 2022 (15)** | **Yes 1** | **Yes 2** | **No** | **No** | **Pyridostigmine** | **escalation** | **recover with successful discharge** | **HAP, respiratory failure** |
| **Baptista et al. 2025 (16)** | **Yes 1** | **Yes 2,3** | **No** | **No** | **Pyridostigmine** | **escalation** | **recover with successful discharge** | **respiratory failure** |
| **Basnet et al. 2024 (17)** | **Yes 1** | **No** | **No** | **No** | **No** | **mono** | **in-hospital death** | **respiratory failure, MOF** |
| **Bawek et al. 2021 (18)** | **Yes 2** | **Yes 1** | **No** | **No** | **Pyridostigmine** | **escalation** | **in-hospital death** | **respiratory failure, heparin induced thrombocytopenia** |
| **Cham et al.2021 (19)** | **Yes 1** | **Yes 2** | **No** | **No** | **No** | **escalation** | **sequelae at follow-up** | **respiratory failure** |
| **Chen et al. 2021 (20)** | **Yes 1** | **No** | **No** | **No** | **No** | **mono** | **in-hospital death** | **abdominal sepsis** |
| **Duong et al. 2021 (21)** | **Yes 1** | **Yes 2** | **No** | **No** | **Pyridostigmine** | **escalation** | **sequelae at follow-up** |  |
| **Fazal et al. 2020 (22)** | **Yes 2** | **Yes 1** | **No** | **No** | **No** | **combination** | **in-hospital death** | **respiratory failure, upper GI bleeding** |
| **Fazel et al. 2019 (23)** | **Yes 1** | **Yes 2** | **No** | **No** | **No** | **escalation** | **in-hospital death** | **respiratory failure** |
| **Fuentes-Antrás et al. 2022 (24)** | **Yes 1** | **Yes 3** | **No** | **Yes 2** | **Pyridostigmine** | **escalation** | **in-hospital death** | **respiratory failure** |
| **Giglio et al. 2020 (25)** | **Yes 1** | **Yes 2** | **No** | **Yes 3** | **No** | **escalation** | **in-hospital death** | **respiratory failure** |
| **Giovannini et al 2023 (26)** | **Yes 1** | **No** | **No** | **No** | **Pyridostigmine** | **escalation** | **in-hospital death** | **respiratory failure** |
| **Hyun et al. 2020 (27)** | **Yes 1** | **No** | **No** | **No** | **No** | **mono** | **in-hospital death** | **respiratory failure** |
| **Hyun et al. 2023 (28)** | **Yes 1** | **No** | **No** | **No** | **No** | **mono** | **in-hospital death** |  |
|  | **Yes 1** | **Yes 2** | **No** | **No** | **No** | **escalation** | **recover with successful discharge** |  |
| **Jenkins et al. 2022 (3)** | **Yes 1** | **Yes 2** | **No** | **No** | **Neostigmine** | **combination** | **recover with successful discharge** |  |
|  | **Yes 1** | **Yes 2** | **No** | **No** | **No** | **combination** | **recover with successful discharge** | **ir pneumonitis** |
| **Jespersen et al. 2021 (29)** | **Yes 1** | **No** | **Yes 3** | **Yes 2** | **No** | **escalation** | **recover with successful discharge** |  |
| **Jeyakumar et al.2020 (30)** | **Yes 1** | **Yes 2,3** | **No** | **No** | **No** | **escalation** | **in-hospital death** | **hyperkalliemia with cardiac arrest** |
| **Kamikawa et al.2025 (31)** | **Yes 2** | **Yes 1,3,5** | **Yes 4** | **No** | **No** | **escalation** | **in-hospital death** | **pneumonia, renal failure** |
| **Karibea et al. 2025 (32)** | **Yes 1** | **No** | **No** | **No** | **No** | **mono** | **recover with successful discharge** |  |
| **Kimura et al. 2016 (33)** | **Yes 1** | **Yes 2,3** | **No** | **No** | **Pyridostigmine** | **escalation** | **recover with successful discharge** |  |
| **Komatsu et al. 2021 (34)** | **Yes 1** | **Yes 2,3** | **No** | **No** | **No** | **escalation** | **in-hospital death** | **respiratory failure** |
| **Konstantina et al. 2019 (35)** | **Yes 1** | **Yes 2** | **Yes 3** | **No** | **Pyridostigmine** | **escalation** | **in-hospital death** | **respiratory failure, septic shock** |
| **Lin et al 2023 (36)** | **Yes 1** | **Yes 2** | **No** | **No** | **Pyridostigmine** | **combination** | **recover with successful discharge** | **respiratory failure** |
| **Lipe et al. 2021 (37)** | **Yes 1** | **Yes 2,3** | **No** | **No** | **No** | **escalation** | **recover with successful discharge** |  |
|  | **Yes 1** | **Yes 2** | **Yes 3** | **No** | **No** | **escalation** | **recover with successful discharge** |  |
|  | **Yes 1** | **Yes 3** | **No** | **Yes 2** | **No** | **escalation** | **in-hospital death** |  |
|  | **Yes 1** | **Yes 4** | **Yes 3** | **Yes 2** | **No** | **escalation** | **recover with successful discharge** |  |
|  | **Yes 1** | **Yes 2** | **No** | **No** | **No** | **escalation** | **recover with successful discharge** |  |
|  | **Yes 1** | **Yes 4** | **Yes 3** | **Yes 2** | **No** | **escalation** | **recover with successful discharge** |  |
|  | **Yes 1** | **Yes 3,4** | **No** | **Yes 2** | **No** | **escalation** | **in-hospital death** |  |
| **Luo et al. 2021 (38)** | **Yes 2** | **Yes 1** | **No** | **No** | **Neostigmine** | **escalation** | **sequelae at follow-up** | **respiratory failure** |
| **Marco et al. 2022 (39)** | **Yes 1** | **Yes 2,3** | **No** | **No** | **No** | **escalation** | **in-hospital death** | **respiratory failure** |
|  | **Yes 1** | **Yes 2,3** | **Yes 4** | **No** | **No** | **escalation** | **in-hospital death** | **respiratory failure** |
|  | **Yes 1** | **Yes 2** | **Yes 3** | **No** | **No** | **escalation** | **in-hospital death** | **respiratory failure** |
|  | **Yes 1** | **Yes 2** | **No** | **No** | **No** | **escalation** | **in-hospital death** |  |
| **Mariniello et al. 2024 (40)** | **Yes 1** | **Yes 2** | **No** | **No** | **Pyridostigmine** | **combination** | **recover with successful discharge** |  |
| **Muranova et al. 2023 (41)** | **Yes 1** | **Yes 2,3** | **Yes 4** | **No** | **Pyridostigmine** | **escalation** | **in-hospital death** | **respiratory failure, septic shock** |
| **Nelke et al. 2023 (42)** | **Yes 1** | **No** | **No** | **Yes 2** | **No** | **escalation** | **recover with successful discharge** |  |
| **Newman et al. 2024 (43)** | **Yes 1** | **Yes 2** | **Yes 3** | **No** | **Pyridostigmine** | **escalation** | **in-hospital death** | **respiratory failure** |
| **Portolés Hernández et al. 2021 (44)** | **Yes 1** | **Yes 3** | **No** | **Yes 2** | **Pyridostigmine** | **escalation** | **in-hospital death** | **respiratory failure** |
| **Pourhassan et al. 2019 (45)** | **Yes 1** | **No** | **No** | **No** | **No** | **mono** | **in-hospital death** | **rabdomyolisis, ir thyroiditis** |
| **Rhee et al. 2022 (46)** | **Yes 1** | **Yes 2** | **No** | **No** | **Pyridostigmine** | **escalation** | **recover with successful discharge** | **respiratory failure** |
| **Rossi et al. 2023 (6)** | **Yes 1** | **No** | **No** | **No** | **No** | **mono** | **in-hospital death** |  |
|  | **Yes 1** | **No** | **No** | **No** | **No** | **mono** | **in-hospital death** | **respiratory failure** |
|  | **Yes 1** | **Yes 2** | **No** | **No** | **No** | **escalation** | **in-hospital death** | **respiratory failure** |
| **Rota et al. 2018 (47)** | **Yes 2** | **Yes 1** | **No** | **No** | **No** | **combination** | **recover with successful discharge** |  |
|  | **Yes 2** | **Yes 1** | **No** | **No** | **No** | **combination** | **in-hospital death** |  |
| **Saishu et al. 2022 (48)** | **Yes 2** | **Yes 1,3** | **No** | **No** | **No** | **combination** | **sequelae at follow-up** | **respiratory failure** |
| **Saito et al. 2024 (49)** | **Yes 1** | **Yes 2,3** | **No** | **No** | **No** | **escalation** | **recover with successful discharge** | **respiratory failure** |
| **Sánchez-Camacho et al. 2025 (50)** | **Yes 1** | **Yes 2,4** | **No** | **Yes 3** | **Pyridostigmine** | **escalation** | **in-hospital death** | **respiratory failure** |
|  | **Yes 1** | **Yes 2** | **No** | **Yes 3** | **Pyridostigmine** | **escalation** | **recover with successful discharge** |  |
|  | **Yes 1** | **Yes 2,3** | **Yes 4** | **No** | **Pyridostigmine** | **escalation** | **sequelae at follow-up** |  |
|  | **Yes 1** | **Yes 3** | **No** | **Yes 2** | **No** | **escalation** | **recover with successful discharge** | **respiratory failure** |
| **Sanchez-Sancho et al. 2021 (51)** | **Yes 1** | **Yes 2** | **Yes 3** | **No** | **No** | **escalation** | **sequelae at follow-up** | **respiratory failure** |
| **Schiopu et al. 2021 (52)** | **Yes 1** | **Yes 2** | **No** | **No** | **No** | **escalation** | **recover with successful discharge** | **respiratory failure** |
| **Schwab et al 2022 (53)** | **Yes 1** | **Yes 2,3** | **Yes 4** | **No** | **Pyridostigmine** | **escalation** | **sequelae at follow-up** | **respiratory failure** |
| **Seker et al. 2023 (54)** | **Yes 1** | **Yes 2,3** | **Yes 4** | **No** | **Pyridostigmine** | **combination** | **recover with successful discharge** | **respiratory failure** |
| **Shibuya et al. 2024 (55)** | **Yes 2** | **Yes 1,3** | **No** | **No** | **No** | **escalation** | **sequelae at follow-up** | **respiratory failure** |
| **Shirai et al. 2019 (56)** | **Yes 1** | **Yes 2** | **No** | **No** | **No** | **combination** | **recover with successful discharge** |  |
| **So et al. 2019 (57)** | **Yes 2** | **Yes 1,3** | **No** | **No** | **No** | **escalation** | **sequelae at follow-up** | **respiratory failure** |
| **Takai et al. 2020 (58)** | **Yes 1** | **Yes 2** | **No** | **No** | **No** | **escalation** | **in-hospital death** |  |
| **Todo et al. 2020 (59)** | **Yes 1** | **No** | **No** | **No** | **No** | **mono** | **recover with successful discharge** | **diarrhea, erythema multiforme** |
| **Veccia et al. 2020 (60)** | **Yes 1** | **Yes 2** | **No** | **No** | **Pyridostigmine** | **escalation** | **in-hospital death** | **respiratory failure** |
| **Vermeulen et al. 2020 (61)** | **Yes 1** | **Yes 2** | **No** | **No** | **Pyridostigmine** | **escalation** | **recover with successful discharge** |  |
| **Wang et al. 2023 (62)** | **Yes 1** | **Yes 2,3** | **No** | **No** | **No** | **escalation** | **in-hospital death** |  |
| **Wang et al. 2022 (63)** | **Yes 1** | **Yes 2** | **No** | **No** | **No** | **combination** | **sequelae at follow-up** | **respiratory failure** |
| **Weaver et al. 2023 (64)** | **Yes 1** | **Yes 2** | **Yes 3** | **No** | **Pyridostigmine** | **escalation** | **recover with successful discharge** | **MG relapse** |
|  | **Yes 1** | **No** | **Yes 2** | **No** | **Pyridostigmine** | **escalation** | **recover with successful discharge** |  |
|  | **Yes 1** | **Yes 2** | **Yes 3,5** | **Yes 4** | **Pyridostigmine** | **escalation** | **recover with successful discharge** | **respiratory failure** |
|  | **Yes 1** | **Yes 2,6** | **Yes 3,5** | **Yes 4** | **Pyridostigmine** | **escalation** | **in-hospital death** | **respiratory failure** |
|  | **Yes 1** | **Yes 2** | **Yes 3** | **No** | **Pyridostigmine** | **escalation** | **recover with successful discharge** |  |
|  | **Yes 1** | **Yes 2** | **Yes 3** | **No** | **Pyridostigmine** | **escalation** | **recover with successful discharge** |  |
|  | **Yes 1** | **Yes 2** | **Yes 3** | **No** | **Pyridostigmine** | **escalation** | **recover with successful discharge** |  |
|  | **Yes 1** | **Yes 2** | **Yes 3** | **No** | **Pyridostigmine** | **escalation** | **recover with successful discharge** |  |
|  | **Yes 1** | **Yes 2** | **Yes 3** | **No** | **Pyridostigmine** | **escalation** | **recover with successful discharge** | **respiratory failure** |
|  | **Yes 1** | **No** | **Yes 2** | **No** | **Pyridostigmine** | **escalation** | **recover with successful discharge** |  |
| **Xie et al. 2021 (65)** | **Yes 1** | **No** | **No** | **No** | **No** | **mono** | **recover with successful discharge** | **ir pneumonitis** |
| **Xing et al. 2020 (66)** | **Yes 1** | **Yes 2,3** | **No** | **No** | **Pyridostigmine** | **combination** | **sequelae at follow-up** | **respiratory failure** |
| **Yanase et al. 2020 (67)** | **Yes 1** | **Yes 2,3** | **No** | **No** | **No** | **escalation** | **recover with successful discharge** |  |
| **Yin et al. 2022 (68)** | **Yes 1** | **Yes 2** | **Yes 3** | **No** | **No** | **combination** | **recover with successful discharge** |  |
